# Multi-Omics Analysis Reveals Nono–Kcnq2 Regulation of Neuronal Excitability in Chronic Constriction Injury-Induced Neuropathic Pain

**DOI:** 10.34133/research.1366

**Published:** 2026-07-14

**Authors:** Peng Chen, Jing Wu, Shaoshuai Tang, Qian Gong, Chen Wang, Wenjing Wang, Yuanhua Wu, Ting Tang, Ruixi Luo, Zhibing Wu, Zhaoyu Qin, Long Wang

**Affiliations:** ^1^ Qihuang College, Guizhou University of Traditional Chinese Medicine, Guiyang 550025, China.; ^2^ Department of Pain Medicine, The First Affiliated Hospital of Guizhou University of Traditional Chinese Medicine, Guiyang 550025, China.; ^3^ State Key Laboratory of Genetic Engineering, Institutes of Biomedical Sciences, School of Life Sciences, Human Phenome Institute, Fudan University, Shanghai 200032, China.; ^4^ First Clinical Medical School, Guangzhou University of Chinese Medicine, Guangzhou 510405, China.; ^5^ State Key Laboratory of Discovery and Utilization of Functional Components in Traditional Chinese Medicine, Guizhou Medical University, Guiyang 561113, China.; ^6^ Department of Pharmacology, School of Pharmacy, Southwest Medical University, Luzhou 646000, China.

## Abstract

Neuropathic pain, resulting from somatosensory nervous system damage or disease, is a debilitating condition marked by spontaneous pain, hypersensitivity, and sensory abnormalities. Neuropathic pain involves spinal neuronal hyperexcitability, yet its molecular basis remains poorly defined. We utilized a comprehensive multi-omics approach, incorporating proteomics, phosphoproteomics, concatenated tandem array of consensus transcription response elements, and both single-cell and spatial transcriptomics, to chart time-resolved adaptations in the L4 to L6 spinal cord of a chronic constriction injury rat model. Multi-omics revealed remodeling of glutamatergic synapse pathways and identified non-POU (Pit-Oct-Unc) domain-containing octamer binding (Nono) as a down-regulated transcription factor associated with reduced potassium voltage-gated channel subfamily Q member 2 (Kcnq2) expression. Nono and Kcnq2 were co-enriched in neurexin 3-positive and peripherin-positive spinal neurons. Restoring Nono expression up-regulated Kcnq2, enhanced K^+^ outward currents (an effect largely abolished by the Kcnq2/3 blocker XE991), and alleviated pain hypersensitivity. Mechanistically, Nono was enriched at a conserved Kcnq2 promoter region in vivo and enhanced Kcnq2 promoter activity in reporter assays. Together, these findings established a Nono–Kcnq2 transcriptional axis that constrained spinal excitability and suggested a therapeutic entry point for neuropathic pain.

## Introduction

Neuropathic pain, resulting from somatosensory nervous system damage or disease, is a debilitating condition marked by spontaneous pain, hypersensitivity, and sensory abnormalities [1]. Neuropathic pain impacts 6.9% to 10% of the global population, leading to substantial physical discomfort and emotional disorders, thereby greatly diminishing quality of life [2,3]. Consequently, neuropathic pain has become a significant global health challenge, posing a considerable burden on healthcare systems around the world. While current pharmacological treatments, including antidepressants, anticonvulsants, opioids, local anesthetics, and nonsteroidal anti-inflammatory drugs, offer only temporary relief by modulating nociceptive signaling, their efficacy is frequently compromised by adverse effects, including tolerance, psychological dependence, and neurotoxicity [4]. As a result, effective management of neuropathic pain remains a formidable challenge, largely due to the incomplete understanding of its complex pathophysiology.

Neuropathic pain is underpinned by intricate peripheral and central mechanisms. The spinal cord serves as a key relay for nociceptive transmission, where sophisticated crosstalk among neurons, glial cells, and supporting elements drives pain initiation and perpetuation [5–7]. Peripheral nerve injury leads to heightened presynaptic glutamate release, which activates postsynaptic α-amino-3-hydroxy-5-methyl-4-isoxazolepropionic acid receptors (AMPARs) and depolarization-dependent *N*-methyl-d-aspartate receptors (NMDARs). This triggers calcium-dependent signaling pathways and extracellular signal-regulated kinase (ERK) activation, enhancing synaptic efficiency and long-term potentiation, essential for spinal sensitization [8,9]. Microglia, the spinal cord’s resident immune cells, undergo profound morphological, transcriptional, and functional changes upon activation, releasing pro-nociceptive and pro-inflammatory mediators that enhance spinal nociceptive circuit activity [10]. The interaction between neurons and glial cells forms a maladaptive pain circuit responsible for the development and continuation of neuropathic pain. This process significantly alters transcription factors (TFs), such as cAMP response element-binding protein, activating transcription factor 3 (ATF3), and nuclear factor of activated T cells, which control gene expression and influence pain signal processing and transmission [11–13].

Despite substantial progress in elucidating the molecular basis of neuropathic pain, the intricate interplay between diverse cell types and their regulatory networks remains largely uncharted. Single-cell RNA sequencing (scRNA-seq) has revolutionized our comprehension of disease mechanisms by enabling the identification of rare or novel cell populations and their gene expression profiles [14]. Although scRNA-seq is increasingly utilized in neuropathic pain research, most studies to date have centered on peripheral nerves, leaving spinal neurons largely unexplored [12,15]. Furthermore, previous scRNA-seq studies on pain have often overlooked spatial information, limiting our ability to decipher the complex spatial and functional organization of pain-related cellular interactions [16]. Spatial transcriptomics, which integrates tissue sectioning with high-throughput sequencing, preserves spatial tissue architecture while mapping gene expression, offering unparalleled insights into cellular diversity, tissue organization, and pathological changes of complex diseases [17,18]. Integrating scRNA-seq with spatial transcriptomics facilitates an in-depth examination of cellular distribution, tissue heterogeneity, and cell–cell interactions within their native spatial environment [19,20]. For instance, the BRAIN Initiative Cell Census Network employed this integrated method to create a comprehensive cell atlas of the mouse brain, elucidating the molecular features and interactions of brain cell types, with profound implications for neurological disease research, including neuropathic pain [20].

This study utilized a multi-omics strategy, combining scRNA-seq, spatial transcriptomics, proteomics, phosphoproteomics, and concatenated tandem array of consensus transcription response elements (catTFRE) analysis, to comprehensively investigate the cellular and molecular changes in the spinal cord of a neuropathic pain rat model. By combining these advanced techniques, we aimed to uncover key cellular and molecular mechanisms driving the development and maintenance of neuropathic pain. Our findings enhance understanding of neuropathic pain pathophysiology and may guide the creation of improved therapeutic approaches.

## Results

### Integrated multi-omics approach unveiled molecular characterization of neuropathic pain in the CCI model

**Fig. 1.**
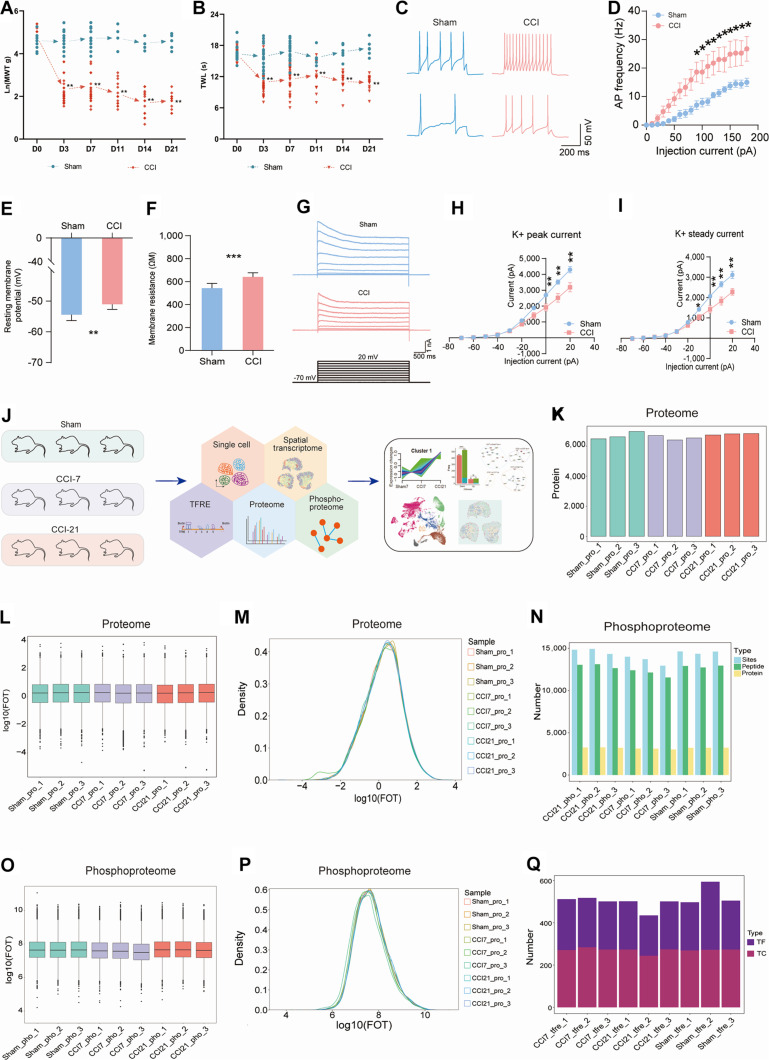
Pain-related behaviors and the multi-omics approach process. (A and B) Mechanical withdrawal threshold (MWT) and thermal withdrawal latency (TWL) values in the chronic constriction injury (CCI) and sham groups at baseline and on postoperative days 3, 7, 11, 15, and 21. The sample sizes were *n* = 30 for the CCI group and *n* = 23 for the sham group at baseline and on postoperative days 3 and 7. After behavioral testing on postoperative day 7, 15 rats per group were randomly sacrificed for L4 to L6 spinal cord collection for multi-omics analyses, leaving *n* = 15 CCI rats and *n* = 8 sham rats for behavioral testing on postoperative days 11, 15, and 21. Each data point represents one individual animal. **P < 0.01 vs. the sham group. (C) Representative action potential firing traces of spinal dorsal horn neurons in sham and CCI groups (*n* = 8 neurons per group). (D) Action potential firing frequency of spinal dorsal horn neurons between sham and CCI groups in response to depolarizing current injections (0 to 180 pA, 500 ms, *n* = 8 neurons per group). (E) Resting membrane potential of spinal dorsal horn neurons in sham and CCI groups (*n* = 8 neurons per group). (F) Membrane resistance of spinal dorsal horn neurons in sham and CCI groups (*n* = 8 neurons per group). (G) Representative K^+^ current traces of spinal dorsal horn neurons in sham and CCI groups (*n* = 8 neurons per group). (H and I) Peak and steady K^+^ current amplitudes of spinal dorsal horn neurons in sham and CCI groups evoked by depolarizing voltage steps (from −70 to +20 mV, 4 s, *n* = 8 neurons per group). (J) The process of multi-omics analysis, including scRNA-seq, spatial transcriptomics, concatenated tandem array of consensus transcription response elements (catTFRE), proteomics, and phosphoproteomics. (K) Bar graphs depicting the number of proteins identified in each sample (*n* = 3 per group). (L) Box plots illustrating the distribution of relative abundance for proteins identified in each sample (*n* = 3 per group). (M) Density distribution analysis of protein abundance. (N) Bar graphs depicting the number of phosphorylation sites, phosphopeptides, and phosphorylated proteins identified in each sample (*n* = 3 per group). (O) Box plots illustrating the distribution of relative abundance for phosphorylated phosphopeptides identified in each sample (*n* = 3 per group). (P) Density distribution analysis of phosphorylated peptide abundance. (Q) Bar graphs showing the number of transcription factors (TFs) and transcription cofactors (TCs) identified in each sample (*n* = 3 per group). **P < 0.01, vs. the sham group.

Mechanical withdrawal threshold (MWT) and thermal withdrawal latency (TWL) were measured at baseline and on days 3, 7, 11, 15, and 21 post-chronic constriction injury (CCI) surgery to assess hyperalgesia. From day 3 post-surgery, MWT and TWL values in CCI model rats significantly decreased (*P* < 0.01) and remained at low levels, indicating marked hyperalgesia (Fig. 1A and B). Neuronal excitability was assessed using whole-cell current-clamp recordings. Neurons from CCI rats exhibited increased firing rates in response to depolarizing current injections relative to the sham group (Fig. 1C). Consistently, the frequency–current curve was shifted upward in the CCI group, indicating increased action potential firing at a given current injection (Fig. 1D). Neurons from CCI rats showed a more depolarized resting membrane potential and increased input resistance (Fig. 1E and F). Outward K^+^ currents were recorded from ipsilateral spinal dorsal horn (SDH) neurons using whole-cell voltage clamp. Depolarizing voltage steps (−70 to +20 mV, 4 s) evoked positive currents (outward, plotted upward), which were reduced in neurons from CCI rats. Analysis of representative traces and quantification indicated a reduction in both peak and steady-state K^+^ current of the CCI group relative to sham controls (Fig. 1G to I). The findings indicated that CCI enhanced the excitability of SDH neurons, potentially due to decreased outward K^+^ currents.

A comprehensive multi-omics analysis, including scRNA-seq, spatial transcriptomics, catTFRE, proteomics, and phosphoproteomics, was performed on spinal cord tissues from sham, CCI7, and CCI21 groups to investigate the molecular mechanisms of neuropathic pain (Fig. 1J). This integrated approach provided an in-depth characterization of the molecular changes driving neuropathic pain induced by CCI. Proteomics and phosphoproteomics analysis respectively identified a substantial number of proteins, phosphorylation sites, phosphopeptides, and phosphorylated proteins across all samples, with consistent detection across biological replicates (Fig. 1K and N). Box plots showed the distribution of relative protein and phosphopeptide abundance across the sham, CCI7, and CCI21 groups, with consistent patterns observed among biological replicates within each group (Fig. 1L and O). Density distribution analysis further validated the trends in protein and phosphopeptide abundance across the different groups, supporting the reliability and reproducibility of the proteomic and phosphoproteomic data (Fig. 1M and P). catTFRE profiling identified a substantial number of TFs and transcription cofactors (TCs) in spinal cord tissues (Fig. 1Q).

### Proteomic analysis identified aberrant protein expression of glutamatergic synapse in neuropathic pain

**Fig. 2.**
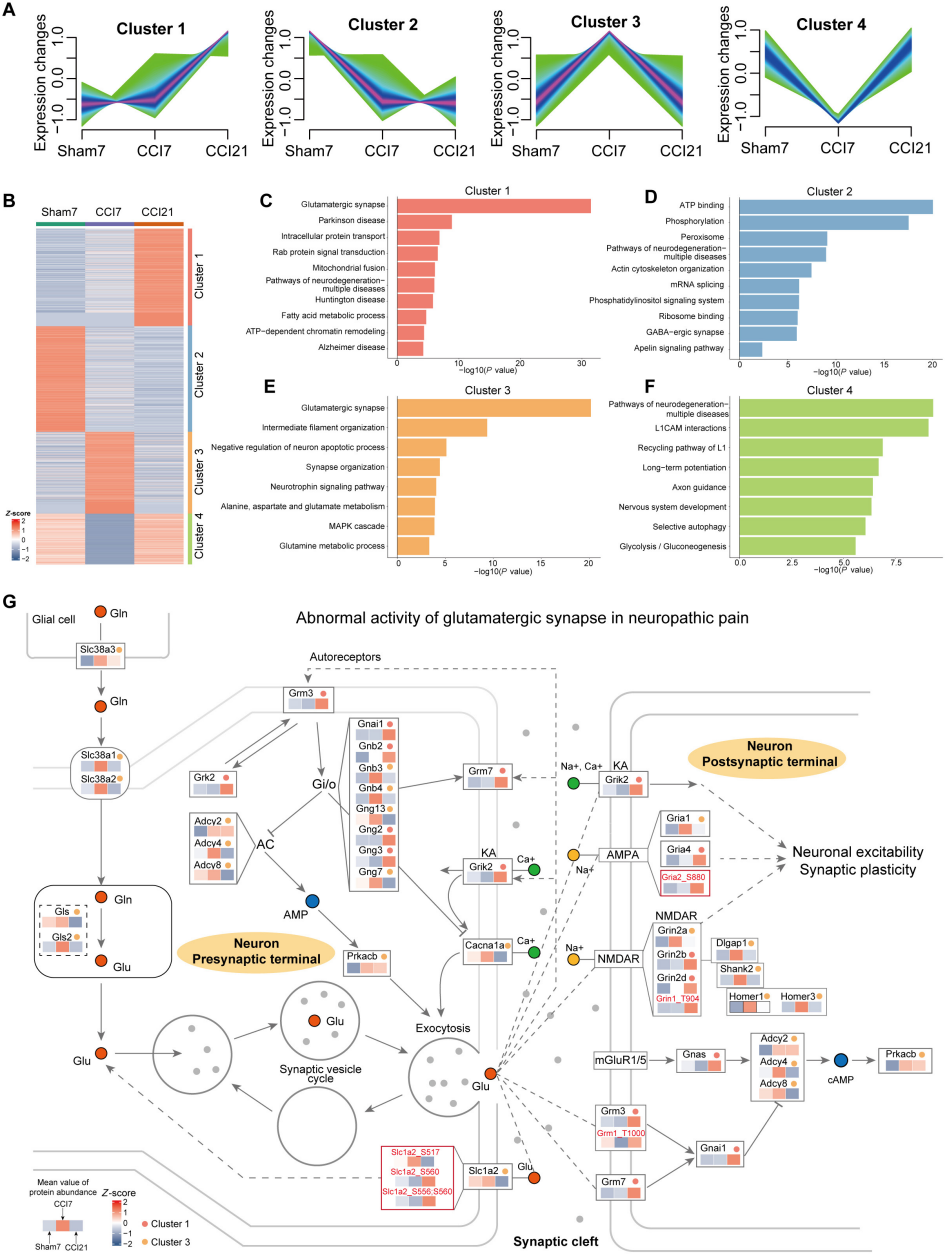
Aberrant glutamatergic neuron activation identified by proteomic analysis in the onset and progression of neuropathic pain. (A) Line charts of protein molecule clusters with distinct expression trends across sham7, CCI7, and CCI21 groups. (B) Heatmap of protein expression in different clusters across sham7, CCI7, and CCI21 groups. (C to F) Database for Annotation, Visualization, and Integrated Discovery (DAVID) pathway enrichment analysis of protein in different clusters. (G) Network diagram of the activated glutamatergic synapse pathway in CCI7 and CCI21.

Proteomics was utilized to detect protein expression changes in the ipsilateral L4 to L6 spinal cord of the neuropathic pain model over 7 to 21 days following CCI. Four distinct protein clusters were identified across sham7, CCI7, and CCI21, each exhibiting unique temporal trends as shown in line plots and a *Z*-score heatmap (Fig. 2A and B). Database for Annotation, Visualization, and Integrated Discovery (DAVID) pathway enrichment analysis identified distinct biological processes and pathways for each cluster (Fig. 2C to F). Cluster 1 was consistently up-regulated at both CCI7 and CCI21, linked to glutamatergic synapse, Parkinson’s disease, and intracellular protein transport. Cluster 2, down-regulated at CCI7 and sustained at low levels through CCI21, was associated with adenosine triphosphate (ATP) binding, phosphorylation, and peroxisome. Cluster 3 peaked at CCI7 before declining, with enrichment in glutamatergic synapse, synapse organization, and neurotrophin signaling pathway. Cluster 4 initially down-regulated at CCI7 with recovery by CCI21, and was linked to neurodevelopment and synaptic plasticity pathways, including long-term potentiation and axon guidance. The signaling pathway diagram encapsulated molecular activities of glutamatergic synapses relevant to neuropathic pain (Fig. 2G). Notable changes were observed at different time points in proteins related to neurotransmitter release (e.g., synaptic vesicle cycle), receptor activity (e.g., NMDARs and AMPARs), and intracellular signaling pathways, suggesting a potential mechanistic link between altered protein expression of glutamatergic synapse and pain progression.

### Phosphoproteomics analysis revealed roles of reduced potassium channel phosphorylation in glutamatergic synapse activation during neuropathic pain

**Fig. 3.**
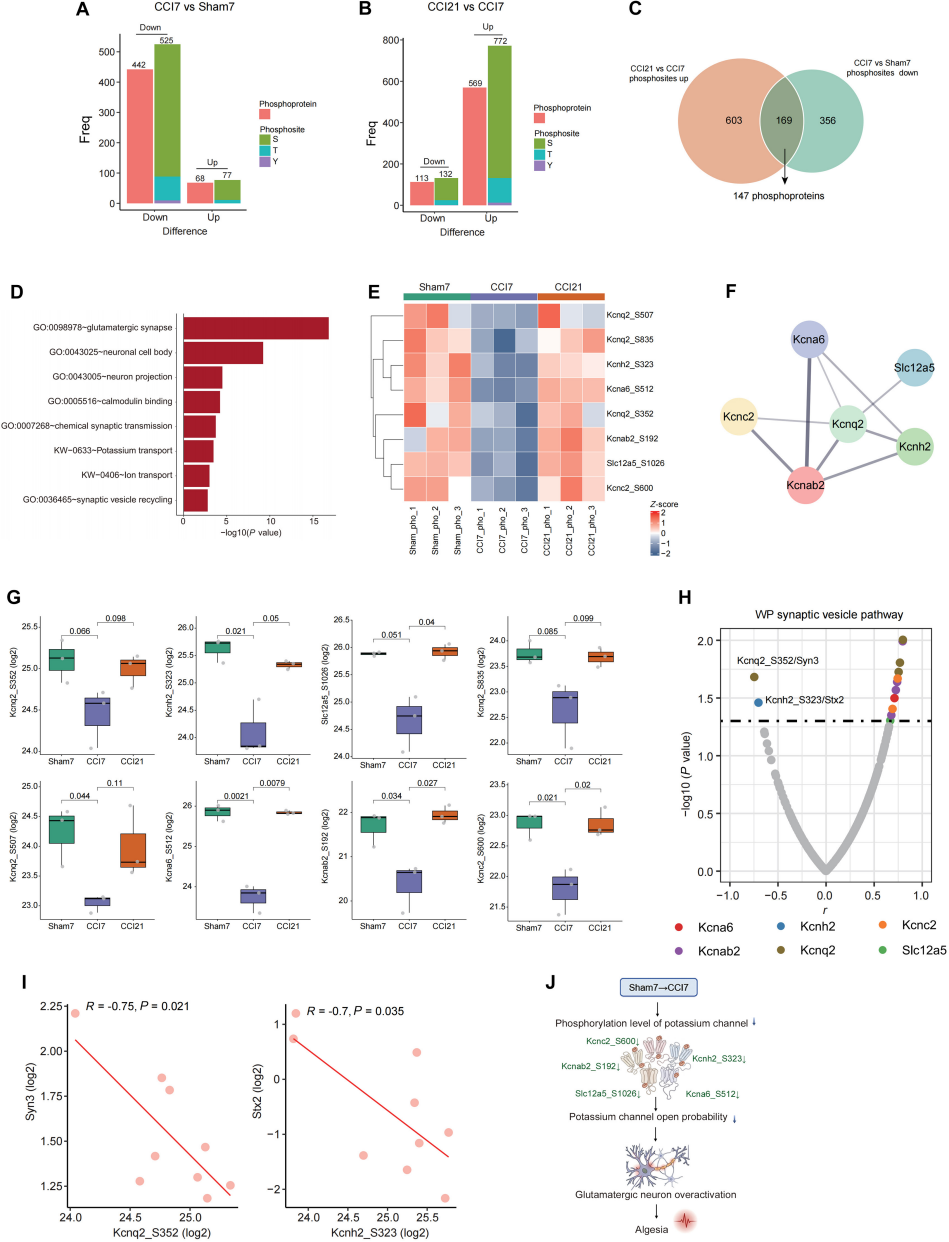
Significant association between reduced phosphorylation of potassium channel proteins and excessive glutamatergic activation revealed by phosphoproteomics. (A and B) Bar graphs illustrating the number of differentially expressed phosphoproteins and phosphosites in CCI7 vs. sham and CCI21 vs. CCI7. (C) Venn diagram depicting the overlap of down-regulated phosphosites in CCI7 vs. sham and up-regulated phosphosites in CCI21 vs. CCI7. (D) Bar graphs illustrating the enriched pathways of down-regulated phosphoproteins in CCI7. (E) Heatmap displaying the expression patterns of phosphorylation sites associated with potassium ion transport pathway across the 3 groups. (F) Interaction network of potassium ion channels. (G) Bar chart of the expression levels in the phosphorylation sites on potassium ion channels. (H) Volcano plot showing the correlation between phosphorylation sites on potassium ion channels and associated molecules involved in the synaptic vesicle pathway. (I) Scatter plot and regression curve of correlations between potassium voltage-gated channel subfamily Q member 2 (Kcnq2)_ S352 and synapsin III (Syn3), as well as potassium voltage-gated channel subfamily H member 2 (Kcnh2)_S323 and syntaxin 2 (Stx2). (J) Flowchart illustrated the relationship between the down-regulated potassium ion channel phosphorylation and excessive glutamatergic neuron activation in CCI7.

Given the essential function of protein phosphorylation in neuronal signaling and spinal sensitization, phosphoproteomics was employed to capture dynamic shifts in spinal protein phosphorylation at specific time points (CCI7 and CCI21), highlighting its regulatory role in neuropathic pain progression. Figure 3A demonstrates a marked decrease in phosphorylation levels of both phosphoproteins and phosphorylation sites (serine, threonine, and tyrosine) at 7 days post-CCI compared to the sham group. In contrast, substantial up-regulation of phosphoproteins and phosphosites was noted at 21 days post-CCI relative to 7 days, with marked increases in serine and threonine phosphorylation (Fig. 3B). The Venn diagram identified 169 overlapping phosphosites down-regulated in CCI7 vs. sham7 and up-regulated in CCI21 vs. CCI7, revealing 147 shared phosphoproteins as potential regulators of early pathological damage or recovery process in neuropathic pain (Fig. 3C). These phosphoproteins mainly participated in the biological process of neuron and synapse, including glutamatergic synapse, neuronal cell body, neuron projection, chemical synaptic transmission, and potassium transport (Fig. 3D). Among these pathways, the abnormal change of phosphorylation level of potassium channel was focused on as the potential pathological mechanism of neuropathic pain. Heatmaps displayed expression changes in potassium channel-related phosphoproteins across the sham7, CCI7, and CCI21, with phosphorylation levels broadly reduced in CCI7 and partially restored in CCI21 (Fig. 3E). The network plot highlighted interactions among potassium channel-associated proteins, such as potassium voltage-gated channel subfamily Q member 2 (Kcnq2), potassium voltage-gated channel subfamily H member 2 (Kcnh2), solute carrier family 12 member 5 (Slc12a5), potassium voltage-gated channel subfamily A member 6 (Kcna6), potassium voltage-gated channel subfamily A regulatory beta subunit 2 (Kcnab2), and potassium voltage-gated channel subfamily C member 2 (Kcnc2) (Fig. 3F), while box plots detailed significant phosphorylation reductions at key sites in CCI7 compared to Sham7, with partial recovery observed by CCI21 (Fig. 3G). Notably, potassium channel-related phosphosites, particularly Kcnq2_S352 and Kcnh2_S323, demonstrated significant involvement in the WikiPathways (WP) synaptic vesicle pathway (Fig. 3H). A strong negative correlation was identified between Kcnq2_S352 and synapsin III (Syn3) expression (*R* = −0.75, *P* = 0.021), as well as between Kcnh2_S323 and syntaxin 2 (Stx2) expression (*R* = −0.70, *P* = 0.035), suggesting a potential involvement of Kcnq2 and Kcnh2 in synaptic vesicle regulation (Fig. 3I). In summary, reduced phosphorylation at potassium channel sites correlates with enhanced pain sensitivity, consistent with known mechanisms linking compromised potassium conductance to neuronal hyperexcitability (Fig. 3J).

### catTFRE analysis indicated down-regulation of Nono TF activity in neuropathic pain

**Fig. 4.**
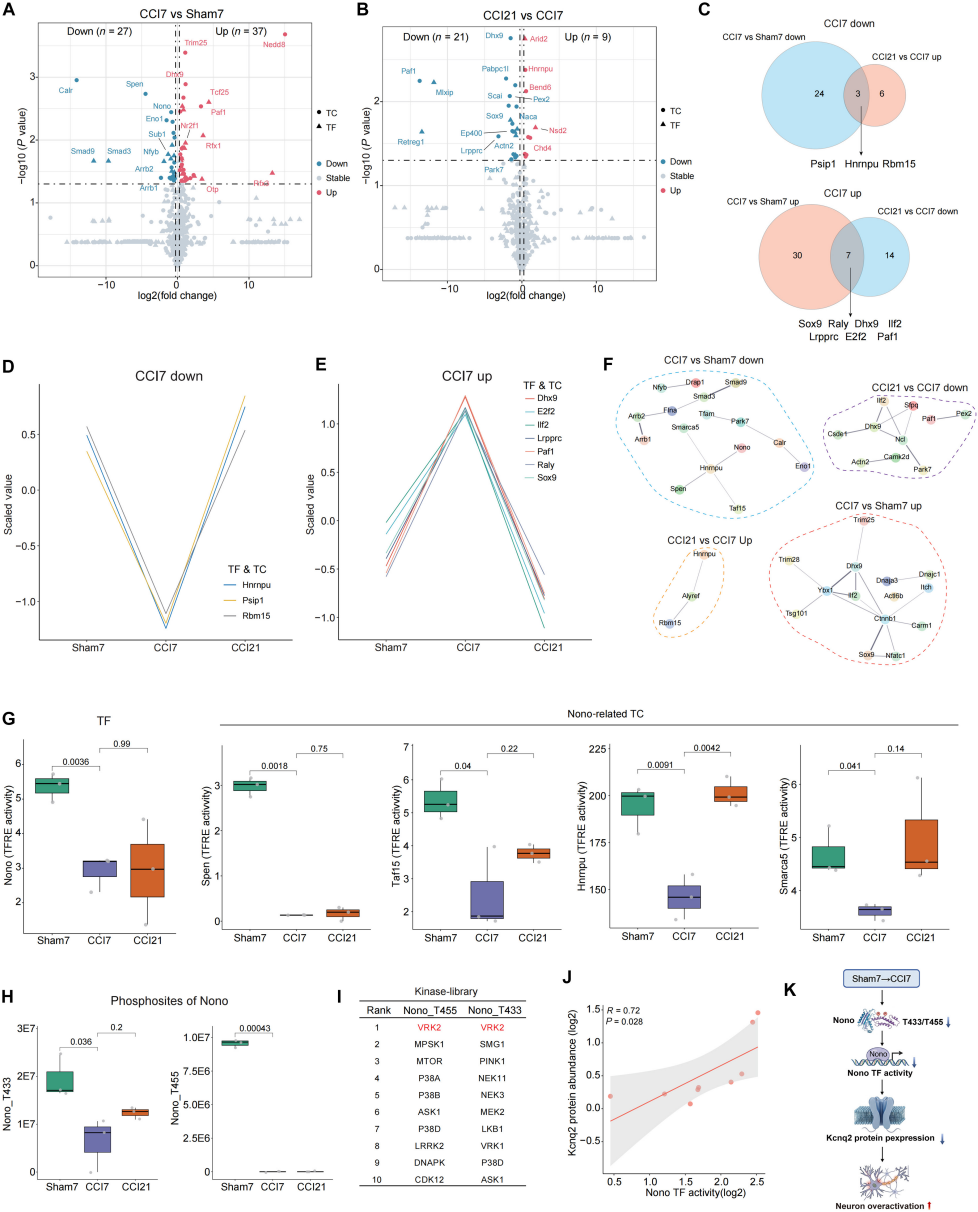
Role of down-regulated non-POU domain containing octamer binding (Nono) activity in neuropathic pain based on catTFRE analysis. (A and B) Volcano plot illustrating differentially expressed TFs and TCs between CCI7 vs. sham7 and CCI21 vs. CCI7. (C to E) Venn diagrams and line charts illustrating the CCI7-specific up-regulation and down-regulation of TFs and TCs in comparison to sham and CCI21 groups. (F) Interaction network of differentially expressed TFs and TCs. (G and H) Bar graph illustrating the differential expression of Nono, its phosphorylation sites, and interacting transcriptional cofactors across the 3 experimental groups. (I) Kinase-library prediction of regulatory kinases for the phosphorylation sites Nono_T455 and Nono_T433. (J) Scatter plot and regression analysis of the correlation between Nono activity and potassium ion channel Kcnq2 expression. (K) Flowchart illustrating the relationship between down-regulated Nono phosphorylation and neuronal hyperactivation in neuropathic pain.

catTFRE analysis was employed to identify alterations in TF activity within the spinal cord during neuropathic pain, highlighting their role in regulating glutamatergic excitability. Seven days after CCI, the spinal cord’s TFs and TCs showed notable alterations relative to the sham group, with 37 genes up-regulated, such as neural precursor cell expressed, developmentally down-regulated 8 (Nedd8), tripartite motif-containing 25 (Trim25), and transcription factor 25 (Tcf25), and 27 genes down-regulated, including non-POU (Pit-Oct-Unc) domain-containing octamer binding (Nono) and spen family transcriptional repressor (Spen) (Fig. 4A). At 21 days post-CCI compared to 7 days, 9 genes were up-regulated, including AT-rich interaction domain 2 (Arid2), heterogeneous nuclear ribonucleoprotein U (Hnrnpu), and nuclear receptor binding SET domain protein 2 (Nsd2), while 21 genes, such as DExH-box helicase 9 (Dhx9) and MLX interacting protein (Mlxip), were down-regulated (Fig. 4B). Six clusters illustrated distinct temporal expression patterns across sham7, CCI7, and CCI21, with line plots depicting trends over time and heatmaps using *Z*-scores to emphasize expression levels (Fig. S1A). Venn diagrams identified 10 shared differentially expressed TFs and TCs between CCI7 vs. sham7 and CCI21 vs. CCI7 (Fig. 4C). These shared factors exhibited distinct expression dynamics across sham, CCI7, and CCI21. Hnrnpu, PC4 and SRSF1 interacting protein 1 (Psip1), and RNA binding motif protein 15 (Rbm15) were down-regulated in CCI7 and then recovered at CCI21, forming a V-shaped pattern. In contrast, Dhx9, E2F transcription factor 2 (E2f2), interleukin enhancer binding factor 2 (Ilf2), leucine-rich pentatricopeptide repeat containing (Lrpprc), and SRY-box transcription factor 9 (Sox9) were up-regulated in CCI7 and then decreased at CCI21, following an inverted V-shaped trend, which indicated dynamic regulation during neuropathic pain progression (Fig. 4D and E). Interaction networks depicted the dynamic regulatory relationships of differentially expressed TFs and TCs between CCI7 vs. sham7 and CCI21 vs. CCI7 comparisons (Fig. 4F).

To further elucidate the regulatory dynamics of these TFs and TCs, we conducted an in-depth analysis of their activities. Nono activity significantly decreased at 7 days post-CCI and remained reduced through 21 days (*P* < 0.05), alongside a similar down-regulation of interacting TCs, such as Spen, Taf15, Hnrnpu, and Smarca5 (Fig. 4G). Phosphorylation levels of Nono at T433 and T455 were significantly decreased at 7 and 21 days post-CCI (*P* < 0.05), indicating Nono’s potential role in modulating pain perception during neuropathic pain progression (Fig. 4H). Kinase-library analysis identified vaccinia-related kinase 2 (VRK2) as the top candidate kinase for phosphorylating both T455 and T433 of Nono, indicating that VRK2 might act as a central regulator of Nono phosphorylation (Fig. 4I). A significant positive correlation was identified between Nono TF activity and Kcnq2 protein abundance through scatter plot and regression analysis (*R* = 0.72, *P* = 0.028; Fig. 4J). Potential regulatory motifs for Nono were identified within the 2-kb upstream region of the Kcnq2 gene, with sites marked by score, strand orientation, and sequence patterns, suggesting possible Nono binding sites implicated in Kcnq2 transcriptional regulation (Fig. S1B and C). In summary, CCI reduced phosphorylation of Nono at T433/T455, which led to diminished TF activity, decreased Kcnq2 protein expression, and ultimately neuronal overactivation (Fig. 4K).

### ScRNA-seq elucidated impact of spinal cord cellular heterogeneity on neuropathic pain

**Fig. 5.**
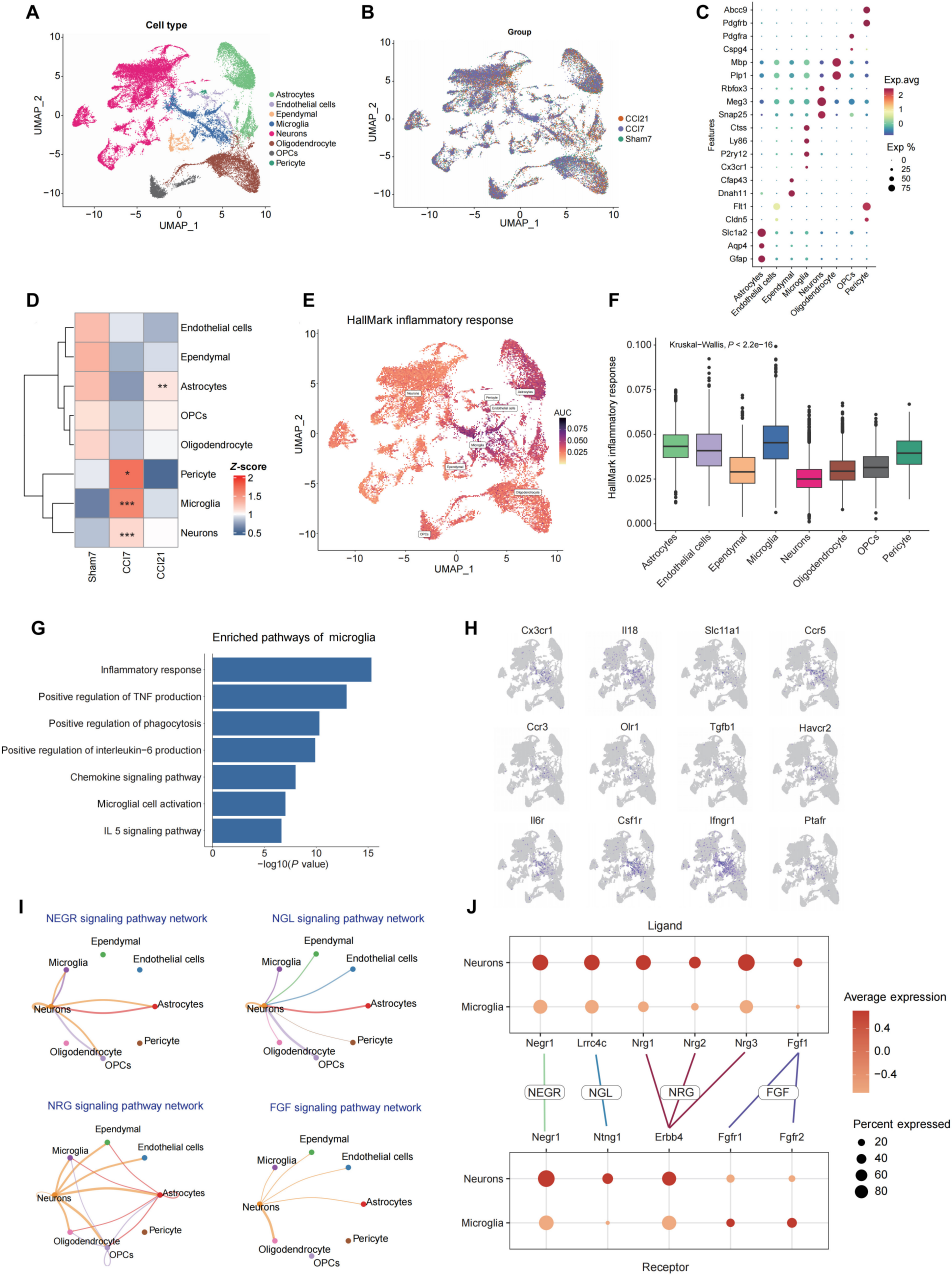
Neuron–glia interactions in neuropathic pain elucidated by scRNA-seq. (A) Uniform manifold approximation and projection (UMAP) plot depicting the distribution of different cell types. (B) UMAP plot depicting the sample distribution across the different groups. (C) Molecular markers of cell types. (D) Heatmaps illustrating the distributional preferences of each cell type across the 3 groups. (E and F) UMAP plot and boxplot showing the inflammatory response levels of different cell types. (G) Enrichment analysis of microglia-related pathways. (H) UMAP visualization of inflammation-related cytokine and receptor expression in microglia. (I and J) CellChat analysis of intercellular communication.

We analyzed cell types in the L4 to L6 spinal cord on days 7 and 21 post-CCI to create a comprehensive atlas of cell-type-specific responses and identify related biological changes. An extensive study of 48,279 high-quality cells identified 8 major cell types based on marker genes: neurons, astrocytes, microglia, oligodendrocytes, oligodendrocyte precursor cells (OPCs), endothelial cells, ependymal cells, and pericytes (Fig. 5A). The uniform manifold approximation and projection (UMAP) visualization depicted the distribution of cell types across different groups (Fig. 5B). Bubble charts showed the molecular markers of different cell types (Fig. 5C). Heatmaps demonstrated the distribution patterns of each cell type across groups, showing a significant increase in microglia and neuron populations at 7 days post-CCI compared to the sham group, and a notable rise in astrocytes at 21 days post-CCI compared to CCI7 (Fig. 5D). UMAP analysis identified notable variations in inflammatory responses across cell types, particularly highlighting increased activity in microglia, pericytes, and astrocytes (Kruskal–Wallis test, *P* < 2.2e−16, Fig. 5E and F). DAVID pathway enrichment analysis revealed significant activation of microglial pathways, including inflammatory response, positive regulation of tumor necrosis factor (TNF) production, interleukin-6 production, phagocytosis, and chemokine signaling pathway, underscoring microglia’s essential role in neuroinflammation and immune modulation linked to neuropathic pain (Fig. 5G). Furthermore, UMAP analysis revealed expression hotspots within microglia for important inflammation-related cytokines and receptor genes, such as C-X3-C motif chemokine receptor 1 (Cx3cr1), interleukin 18 (Il18), solute carrier family 11 member 1 (Slc11a1), C-C motif chemokine receptor 5 (Ccr5), and oxidized low density lipoprotein receptor 1 (Olr1) (Fig. 5H). CellChat analysis depicted intercellular communication, revealing networks of neuronal growth regulator (NEGR), netrin-G ligand (NGL), neuregulin (NRG), and fibroblast growth factor (FGF) signaling pathways between microglia and neurons (Fig. 5I and J).

**Fig. 6.**
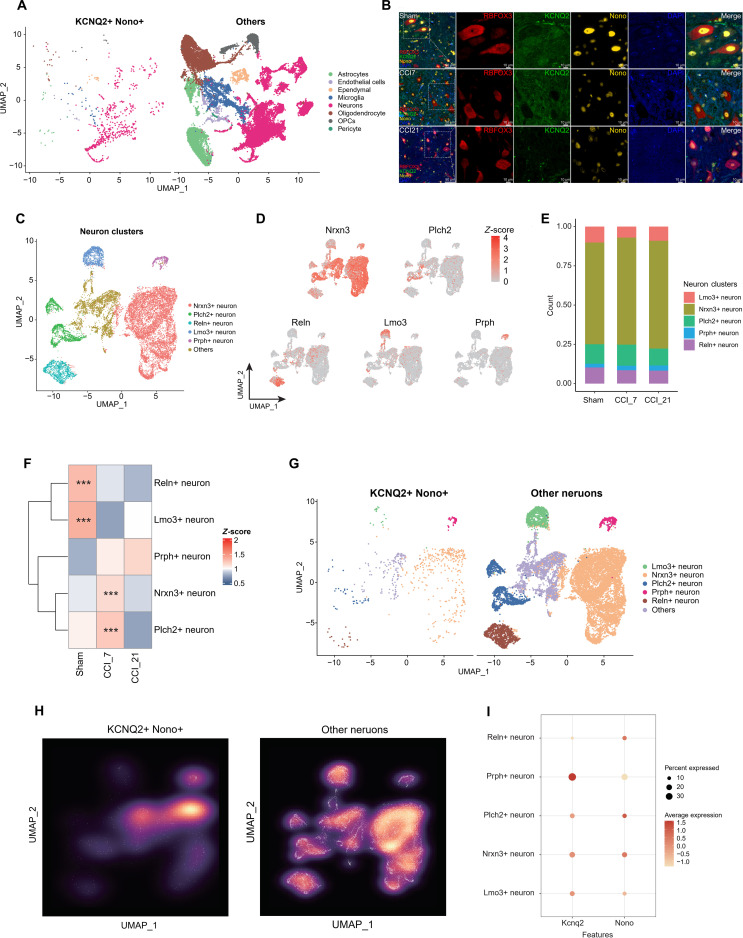
Colocalization of Nono and Kcnq2 in scRNA-seq. (A) UMAP plot showing the colocalization of Nono and Kcnq2 across different cell types. (B) Immunofluorescence analysis confirming the colocalization of Nono and Kcnq2 in neurons. (C) UMAP plot of the distinct neuronal subpopulations. (D) Expression of molecular markers in neuronal subpopulations. (E) Bar graphs showing the proportion of different neuronal subgroups across the 3 groups. (F) Heatmaps illustrating the distributional preferences of neuronal subpopulations. (G and H) Colocalization of Nono and Kcnq2 in the distinct neuronal subpopulations. (I) Expression of Nono and Kcnq2 across the distinct neuronal subpopulations.

The UMAP plot showed that Nono and Kcnq2 primarily colocalize in neurons (Fig. 6A). Immunofluorescence analysis demonstrated the colocalization of Kcnq2 and Nono in neurons, with significantly decreased fluorescence intensity observed at both 7 and 21 days following CCI (Fig. 6B). To elucidate the role of neurons in spinal central sensitization, we conducted a comprehensive analysis to uncover gene expression patterns and molecular mechanisms within distinct neuronal subpopulations. UMAP clustering identified neuronal subpopulations in the spinal cord, each defined by specific marker genes, including neurexin 3 (Nrxn3), phospholipase C eta 2 (Plch2), reelin (Reln), LIM domain only 3 (Lmo3), and peripherin (Prph) (Fig. 6C). The expression patterns of these markers are shown in Fig. 6D, with *Z*-score color-coding indicating high-expression regions in red. Bar charts and heatmaps illustrated the proportions and distributional preferences of each neuronal subtype across different groups, revealing varying levels of increase in Nrxn3^+^, Plch2^+^, and Prph^+^ neurons (Fig. 6E and F). UMAP analysis further demonstrated the colocalization of Nono and Kcnq2 in Prph^+^ and Nrxn3^+^ neurons (Fig. 6G and H). The dot plot showed the expression of Nono and Kcnq2 across different neuronal subtypes, revealing that Nono and Kcnq2 were most abundantly expressed in Nrxn3^+^ and Prph^+^ neurons (Fig. 6I). CellChat analysis depicted intercellular communication among microglia, astrocytes, and various neuronal subtypes through the NRXN, NRG, and neural cell adhesion molecule (NCAM) signaling pathways (Fig. S2A and B).

### Spatial transcriptomics revealed the cellular composition and distribution within the spinal cord in neuropathic pain

**Fig. 7.**
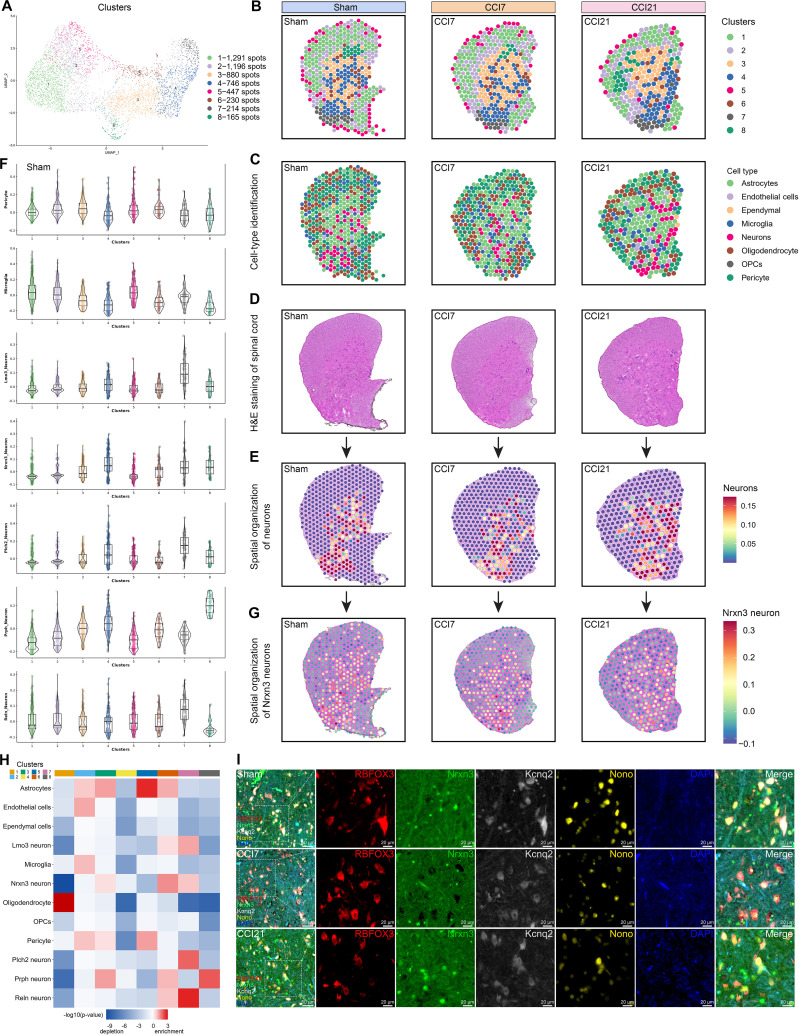
Cellular composition and distribution of the spinal cord in neuropathic pain illustrated by spatial transcriptomics. (A and B) UMAP plot showing the distribution of clusters in spatial transcriptomics. (C) Spatial distribution of different cell types across the sham, CCI7, and CCI21 groups. (D) H&E staining of ipsilateral spinal cord tissues. (E) Spatial neuronal distribution across the sham, CCI7, and CCI21 groups. (F) Violin plot showing expression levels of different cell types across clusters. (G) Spatial distribution of neurexin 3 (Nrxn3)-positive neuron across the sham, CCI7, and CCI21 groups. (H) Multimodal intersection analysis (MIA) showing the colocalization of Nrxn3^+^, peripherin (Prph)-positive neurons, and other cell types across different clusters. (I) Immunofluorescence analysis confirming the colocalization of Nono and Kcnq2 in Nrxn3^+^ neurons.

We conducted spatial transcriptomics on ipsilateral L4 to L6 spinal cord sections from sham, CCI7, and CCI21 rats (*n* = 3 per group) to analyze the spatial distribution of Nono–Kcnq2 coexpressing neurons. This resulted in 1,456, 1,212, and 1,321 spots with median gene counts of 958, 1,040, and 1,183 per spot, respectively. Based on gene expression profiling, we classified the spots into 8 distinct clusters, as visualized by the UMAP plot (Fig. 7A and B and Fig. S3A). We utilized the robust cell-type decomposition method, integrating spatial transcriptomics with scRNA-seq data to estimate cell-type proportions in multicell spots [21]. The spatial transcriptomics tissues identified various cell types, including neurons, microglia, astrocytes, oligodendrocytes, OPCs, endothelial cells, ependymal cells, and pericytes (Fig. 7C and Fig. S3B). Hematoxylin and eosin (H&E) staining identified ipsilateral spinal cord tissue, with neurons predominantly situated in the spinal cord gray matter (Fig. 7D and E and Fig. S3C and D). Through AddModuleScore analysis, the violin plots showed the distribution scores of different neuronal subtypes across 8 distinct clusters (Fig. 7F and Fig. S3E and F). Further, we mapped the spatial distribution of Prph^+^ and Nrxn3^+^ neurons, finding Nrxn3^+^ neurons predominantly in the spinal cord dorsal horn and Prph^+^ neurons in the anterior horn (Fig. 7G and Fig. S3G and H). Compared to the sham group, Prph^+^ neurons in CCI21 and Nrxn3^+^ neurons in CCI7 showed a trend of increased spatial distribution. Multimodal intersection analysis (MIA) was employed to assess the colocalization of Nrxn3^+^ neurons, Prph^+^ neurons, and other cell types across different clusters (Fig. 7H) [22]. Immunofluorescence analysis confirmed that Nrxn3^+^ neurons predominantly resided in the dorsal horn, whereas Prph^+^ neurons were mainly located in the anterior horn. CCI injury notably decreased the coexpression of Nono and Kcnq2 specifically within Nrxn3^+^ and Prph^+^ neurons compared to the sham group (Fig. 7I and Fig. S3I), suggesting that nerve injury disrupts the transcriptional regulation of Kcnq2 in these neuronal populations. These results indicated that distinct neuronal subtypes exhibit differential vulnerability to injury-induced dysregulation of this transcriptional axis.

### Nono overexpression attenuates neuropathic pain by transcriptionally activating Kcnq2 in the spinal cord

**Fig. 8.**
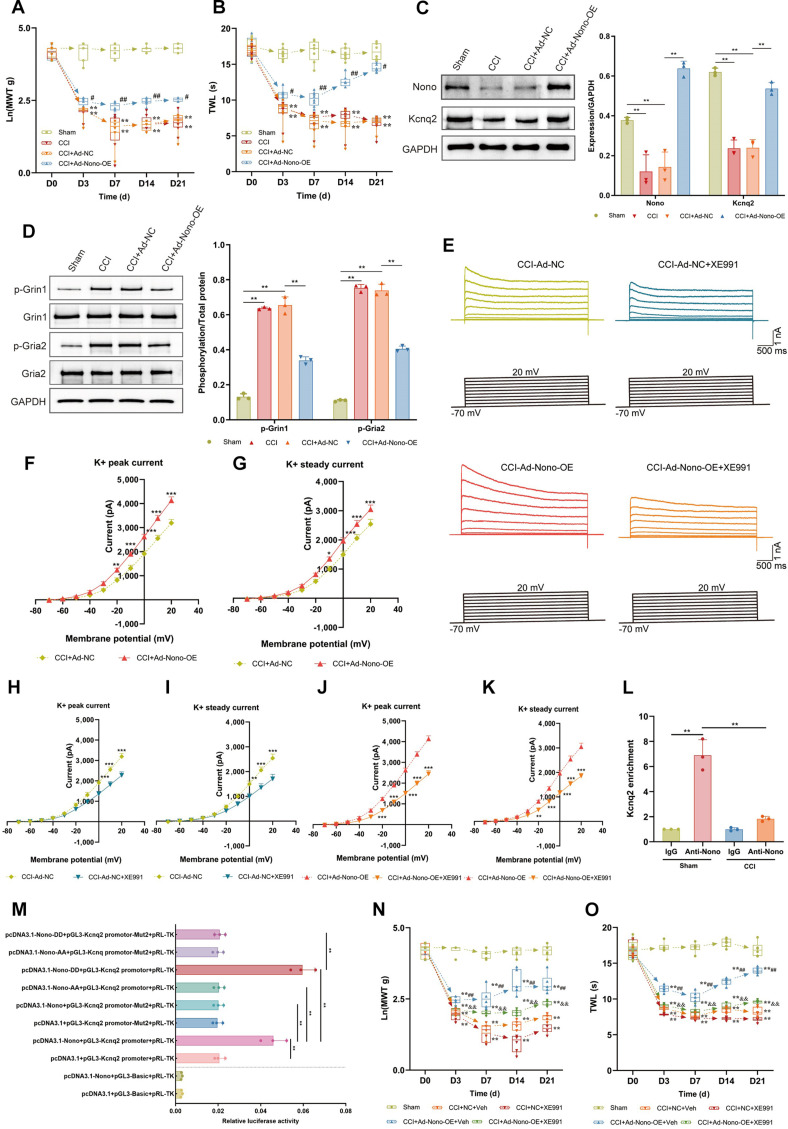
Nono overexpression alleviates neuropathic pain via transcriptional activation of Kcnq2 in the spinal cord. (A and B) Behavioral assessment of MWT and TWL following CCI surgery and intrathecal administration of Nono-expressing adenovirus (CCI+Ad-Nono-OE) or control virus (CCI+Ad-NC, *n* = 8 per group). **P < 0.01vs. the sham group. #*P* < 0.05, ##*P* < 0.01 vs. the CCI+Ad-NC group. (C) Western blot analysis of Nono and Kcnq2 protein expression in L4 to L6 spinal cord tissue collected 21 days after surgery (*n* = 3 per group). (D) Western blot analysis of p-Grin1 and p-Gria2 expression in L4 to L6 spinal cord tissue collected 21 days after surgery (*n* = 3 per group). (E) Representative K^+^ current traces of spinal dorsal horn neurons in CCI+Ad-Nono-OE and CCI+Ad-NC control groups (*n* = 8 neurons per group). (F and G) Peak and steady K^+^ current amplitudes of spinal dorsal horn neurons in CCI+Ad-Nono-OE and CCI+Ad-NC control groups evoked by depolarizing voltage steps (from −70 to +20 mV, 4 s, *n* = 8 neurons per group). (H and I) Peak and steady K^+^ current amplitudes of spinal dorsal horn neurons in CCI+Ad-NC and CCI+Ad-NC+XE991 (Kcnq2/3-M current inhibitor) evoked by depolarizing voltage steps (from −70 to +20 mV, 4 s, *n* = 8 neurons per group). (J and K) Peak and steady K+ current amplitudes of spinal dorsal horn neurons in CCI+Ad-Nono-OE and CCI+Ad-Nono-OE+XE991 evoked by depolarizing voltage steps (from −70 to +20 mV, 4 s, *n* = 8 neurons per group). (L) ChIP assays demonstrating increased Nono binding to the Kcnq2 promoter in the spinal cord. (M) Dual-luciferase reporter assays in HEK-293T cells assessing the effects of Nono and its T433/T455 phosphorylation-site mutants on Kcnq2 promoter-driven luciferase activity. (C to M) *P < 0.05, **P < 0.01, ***P < 0.001 vs. the corresponding control group. (N and O) Behavioral assessment of MWT and TWL showing that XE991 partially reverses the Nono overexpression-induced alleviation in mechanical allodynia and thermal hyperalgesia after CCI surgery (*n* = 6 per group). **P < 0.01 vs. the sham group. ##*P* < 0.01 vs. the CCI+Ad-NC +Vehicle (Veh) group. &&*P* < 0.01 vs. the CCI+Ad-Nono-OE group.

To determine whether Nono plays a causal role in regulating neuropathic pain, we constructed a recombinant adenovirus expressing Nono and delivered it intrathecally into rats following CCI surgery. Nono overexpression significantly increased MWT and TWL from day 3 to day 21 post-surgery, relative to CCI rats treated with control virus (Fig. 8A and B), suggesting a sustained attenuation of mechanical and thermal hypersensitivity. To further verify the molecular effects of Nono overexpression in vivo, spinal cord tissues were collected on postoperative day 21 for Western blot analysis and immunofluorescence staining. CCI markedly reduced Nono and Kcnq2 protein levels compared with sham controls, whereas Nono overexpression restored Nono expression and increased Kcnq2 protein abundance in the ipsilateral L4 to L6 spinal cord (Fig. 8C). To further address whether Nono restoration was observed in the neuronal subtypes implicated by our single-cell and spatial transcriptomic analyses, we performed additional multicolor immunofluorescence staining for RBFOX3, Nono, Kcnq2, and either Nrxn3 or Prph. Compared with the CCI+Ad-NC group, Ad-Nono-OE was associated with increased Nono immunoreactivity in both RBFOX3^+^/Nrxn3^+^ and RBFOX3^+^/Prph^+^ neurons, accompanied by increased Kcnq2 signal in these neuronal populations (Fig. S4A and B). Quantitative analysis confirmed increased mean fluorescence intensity of Nono and Kcnq2 in Nrxn3^+^ and Prph^+^ neurons after Ad-Nono-OE treatment (Fig. S4C to F). Given the key role of postsynaptic glutamatergic signaling in neuropathic pain, we further assessed the phosphorylation levels of Grin1 and Gria2, 2 major postsynaptic subunits of NMDARs and AMPARs, respectively. Nono overexpression significantly decreased the p-Grin1/Grin1 and p-Gria2/Gria2 ratios (Fig. 8D), suggesting that Nono restoration was associated with attenuated phosphorylation of postsynaptic glutamatergic receptor subunits after CCI injury. These findings should be interpreted as supportive observations of altered postsynaptic glutamatergic signaling rather than direct functional validation of specific phosphorylation sites. Whole-cell voltage-clamp recordings from SDH neurons revealed outward K^+^ currents evoked by depolarizing steps from −70 to +20 mV. Compared with the CCI+Ad-NC group, Nono overexpression (CCI+Ad-Nono-OE) produced larger outward currents across depolarized potentials (Fig. 8E to G). XE991, a Kcnq2/3-selective blocker, markedly reduced outward currents and largely eliminated the Nono overexpression-associated increase (Fig. 8H to K) [23]. Chromatin immunoprecipitation (ChIP) assays demonstrated Nono enrichment at a conserved region of the Kcnq2 promoter in spinal cord tissue (Fig. 8L), and dual-luciferase assays further showed that Nono significantly increased Kcnq2 promoter-driven activity (Fig. 8M), supporting Nono-dependent transcriptional activation of Kcnq2 in a reporter assay. To further determine whether Nono phosphorylation at T433/T455 contributes to its transcriptional activity, we generated phospho-deficient Nono-T433A/T455A (Nono-AA) and phospho-mimetic Nono-T433D/T455D (Nono-DD) mutants. In dual-luciferase assays, Nono-DD showed stronger activation of the Kcnq2 promoter than wild-type Nono, whereas Nono-AA markedly reduced promoter activation, indicating that phosphorylation at T433/T455 positively regulates Nono-mediated Kcnq2 promoter activity in the reporter system (Fig. 8M). We next tested whether this transcriptional activation depended on the predicted Nono-binding motif within the Kcnq2 promoter. The core AGTGAG motif, corresponding to the reverse-complement CTCACT motif identified in the motif analysis, was mutated to GACAGA to generate the Kcnq2 promoter-Mut2 reporter. Mutation of this motif substantially reduced basal promoter activity and largely abolished the activation induced by wild-type Nono and Nono-DD (Fig. 8M). These findings indicate that the AGTGAG motif is required for Nono-dependent activation of the Kcnq2 promoter reporter in the dual-luciferase assay and that the phosphorylation-dependent enhancement of Nono transcriptional activity depends on this motif in the reporter system. Behavioral tests were performed to evaluate whether XE991 could reverse the analgesic effect of Nono overexpression after CCI surgery. Compared with the CCI+Ad-Nono-OE group, XE991 treatment significantly reduced MWT and TWL, indicating that blockade of Kcnq2/Kv7 channels partially reversed the analgesic effect of Nono overexpression after CCI surgery (Fig. 8N and O). Together, these results support a model in which CCI-induced reduction of Nono phosphorylation and activity may contribute to decreased Kcnq2 expression and promoter activity, thereby reducing outward K^+^ currents and enhancing spinal neuronal excitability.

## Discussion

The spinal cord is crucial in pain processing and transmission, significantly influencing the progression of pain pathways. Key spinal mechanisms underlying neuropathic pain encompass central sensitization, glial cell activation, excessive excitatory neurotransmitter release, diminished inhibitory neurotransmission, and ion channel dysregulation [10,24]. Collectively, these spinal processes intensify and perpetuate pain signaling, contributing to the amplification and persistence of pain signaling. However, the limited understanding of the cellular and molecular mechanisms governing these processes remains a major barrier to advancing effective pain treatments. In this study, we utilized scRNA-seq, spatial transcriptomics, catTFRE, proteomics, and phosphoproteomics to comprehensively characterize dynamic cellular responses, regulatory networks, and intricate molecular mechanisms underlying spinal processes in neuropathic pain across specific time points.

Central sensitization refers to an intensified functional state of neurons and circuits within nociceptive pathways, marked by increased membrane excitability, enhanced synaptic efficacy and diminished inhibitory signaling [25]. Activation of postsynaptic NMDARs and the insertion of AMPARs into the plasma membrane are essential for inducing and maintaining central sensitization [26]. NMDARs are heterotetrameric, ligand-gated cation channels, consisting of 2 GluN1 subunits paired with either glutamate-binding GluN2 (A to D) or glycine-binding GluN3 (A and B) subunits [27]. The biophysical, pharmacological, and signaling properties of each NMDAR complex are determined by its subunit composition, with GluN2A and GluN2B subtypes acting as key regulators. These subtypes are prominently expressed in the nervous system and vital for nociceptive processing [28–30]. While the role of GluN2A in pain remains uncertain, activation of GluN2B drives substantial calcium influx and downstream signaling cascades that potentiate synaptic plasticity and neurotoxicity, thereby promoting the persistence of neuropathic pain and heightened sensitivity to pain [31–33]. AMPARs are ion channels activated by glutamate, consisting of 4 subunits (GluA1 to GluA4) that form functional tetramers [34]. In pain states, GluA1-containing AMPA receptors are significantly up-regulated, boosting synaptic transmission efficiency, driving synaptic plasticity changes, and elevating neuronal excitability [35,36]. In our study, proteomics and phosphoproteomics analysis identified sustained up-regulation in both protein expression and some key phosphorylation levels of NMDAR subunits GluN2A, GluN2B, and GluN2D, along with AMPAR subunits GluA1 and GluA4, in spinal cord tissue of the CCI model group at both 7 and 21 days post-surgery. Together, these molecular alterations are consistent with robust induction and maintenance of central sensitization in the spinal cord following CCI.

The excessive release of glutamate from spinal presynaptic neurons is a critical driver of spinal cord central sensitization in neuropathic pain, reflecting disruptions in its synthesis, storage, and release pathways [26]. Its synthesis depends largely on the enzymatic conversion of glutamine to glutamate. Extracellular glutamine is actively imported into presynaptic neurons via specific glutamine transporters, such as solute carrier family 38 member 1 (SLC38A1/SNAT1) and solute carrier family 38 member 2 (SLC38A2/SNAT2), where it is subsequently converted into glutamate by glutaminase (GLS) [37]. Proteomic analysis showed a temporary 1.5- to 2-fold increase in SLC38A1 and GLS2 expression in the ipsilateral spinal cord at day 7 following CCI, which returned to baseline by day 21, implicating their roles in the early up-regulation of glutamate synthesis during neuropathic pain. Synthesized glutamate is loaded into synaptic vesicles via vesicular glutamate transporters, with its release being precisely controlled by ion channel function, neuronal excitability, and synaptic signaling [38–40]. Potassium ion channels are essential for the accurate regulation of glutamate release. Numerous studies have shown a reduction of potassium channel activity and expression in several animal models of neuropathic pain [40,41]. Inhibition of these channels increases action potential firing frequency and duration, potentiates Ca^2+^ influx through voltage-gated Ca^2+^ channels, and ultimately facilitates glutamate release [42–44]. Proteomic and phosphoproteomic analyses on day 7 post-CCI revealed a marked decrease in the expression and phosphorylation of several potassium ion channels, such as Kcnq2, Kcna6, and Kcnc2, which displayed strong associations with synaptic proteins. Kcnq2, a critical member of the KCNQ (Kv7) potassium channel family, controls low-threshold, non-inactivating M-currents in the nervous system, acting as a key regulator of neuronal excitability and maintaining the equilibrium of pain signal transmission [45]. In neuropathic pain, Kcnq2 expression and function are profoundly altered, characterized by reduced expression and disrupted channel currents, which contribute to increased synaptic excitability and aggravated pain sensitization [46,47]. Kcnq2 channel activators selectively inhibit A-fiber-mediated glutamate release in the SDH, effectively alleviating mechanical hypersensitivity and other pain behaviors associated with neuropathic pain [48]. This study revealed a substantial reduction in Kcnq2 protein expression to approximately 1/4 to 1/5 of normal levels at 7 and 21 days following CCI, accompanied by a notable down-regulation of several phosphorylation sites. This down-regulation is anticipated to enhance neuronal excitability and amplify the transmission of pain signals. While sustained suppression of Kcnq2 remains evident at CCI21, the partial phosphorylation recovery coincides with adaptive molecular compensations—specifically elevated expression of γ-aminobutyric acid (GABA) receptor subunits—suggesting that system-level homeostatic rebalancing may create permissive conditions for synaptic Kcnq2 stabilization during chronic pain progression, independent of direct transcriptional restoration.

Widespread transcriptional alterations are evident in neuropathic pain, indicating the essential role of TFs in nociceptive processing and transmission, chronic pain development and persistence, and nerve regeneration [49]. Dysregulated TFs, such as nuclear factor κB (NF-κB), interferon regulatory factor 5 (IRF5), and c-Fos, have been shown to drive neuroinflammation, modulate neuronal excitability and synaptic plasticity, and contribute to pain [50–52]. In addition to these classic TFs, our catTFRE analysis and phosphoproteomic profiling identified a marked reduction in both expression and phosphorylation of Nono by day 7 following CCI, with levels remaining consistently suppressed through day 21. Nono is a multifunctional nuclear protein and a member of *Drosophila* behavior/human splicing (DBHS) protein family, which includes its paralogs, paraspeckle component 1 (PSPC1) and splicing factor proline/glutamine rich (SFPQ) [53]. Nono contains an N-terminal RNA recognition motif (RRM) and a C-terminal helix-turn-helix (HTH) domain, facilitating direct interactions with RNA and DNA [54]. As a molecular scaffold, Nono plays a crucial role in gene expression regulation by coordinating transcription, mRNA splicing, RNA retention, and facilitating non-homologous end joining (NHEJ) to maintain genomic stability [54–56]. Recent researches have highlighted Nono’s critical involvement in neuronal development, synaptic regulation, and neurological disorders [57–60]. Our findings, aligned with these studies, emphasized the significance of Nono in the initiation and persistence of neuropathic pain. Furthermore, we identified a positive correlation between Nono activity and Kcnq2 expression, along with the presence of a potential Nono-binding motif within the 2-kb upstream regulatory region of the Kcnq2 gene. This suggested that Nono potentially regulated Kcnq2 expression by interacting with its upstream regulatory elements. However, CCI-induced suppression of Nono activity likely contributes to the down-regulation of Kcnq2 expression, offering insights into its role in pain modulation. Our in vivo gain-of-function experiments, using intrathecal delivery of a Nono-overexpressing adenovirus in CCI rats, indicate that increasing spinal Nono is sufficient to mitigate neuropathic pain. Nono overexpression alleviated mechanical allodynia and thermal hyperalgesia, with elevated MWT and TWL from day 3 to day 21 post-injury. These behavioral effects were accompanied by restoration of spinal Nono and Kcnq2 protein levels, reversing injury-induced reductions. Consistently, whole-cell recordings from dorsal horn neurons showed enhanced depolarization-evoked outward K^+^ currents after Nono overexpression, and the Nono-dependent current increase was largely abolished by the Kcnq2/3-selective blocker XE991, supporting a prominent Kcnq2-associated electrophysiological component. ChIP assays demonstrated Nono’s enrichment at the Kcnq2 promoter, while dual-luciferase reporter assays showed that Nono increased Kcnq2 promoter reporter activity.

Importantly, our additional mutagenesis experiments further strengthened this mechanistic link within the reporter-assay context. The phospho-mimetic Nono-T433D/T455D mutant displayed enhanced transcriptional activation of the Kcnq2 promoter, whereas the phospho-deficient Nono-T433A/T455A mutant showed markedly impaired promoter activation. These findings suggest that phosphorylation at T433/T455 is functionally important for Nono-mediated Kcnq2 promoter reporter activity. Moreover, mutation of the core AGTGAG motif within the Kcnq2 promoter to GACAGA largely abolished the responsiveness of the promoter to both wild-type Nono and phospho-mimetic Nono, indicating that this motif is required for Nono-dependent promoter activation in the reporter assay. However, because these experiments were performed in a heterologous HEK-293T reporter system, they do not demonstrate that the AGTGAG motif is required for endogenous Kcnq2 regulation at the genomic locus in spinal neurons, nor do they establish whether the Nono-T433A/T455A and Nono-T433D/T455D mutants regulate endogenous Kcnq2 mRNA or protein expression in neurons. Future studies using locus-specific perturbation, genome-editing approaches, primary spinal neurons, or other appropriate neuronal models will be needed to address these issues. Together with the in vivo overexpression, protein expression, and electrophysiological data, these findings support the Nono–Kcnq2 axis as a transcriptionally regulated mechanism that modulates potassium channel expression and spinal neuronal excitability. However, the behavioral effects of Nono restoration should be interpreted with appropriate caution. Although Nono overexpression markedly restored Nono and Kcnq2 protein levels and significantly improved pain-related behaviors, MWT and TWL did not fully return to sham levels. This partial behavioral recovery suggests that the Nono–Kcnq2 axis is an important contributor to spinal neuronal hyperexcitability, but may not fully account for the entire pain phenotype after CCI. Therefore, our findings support a more balanced interpretation in which the Nono–Kcnq2 axis acts as a key modulatory pathway rather than the sole determinant of neuropathic pain. This more refined interpretation also provides a rational basis for considering the therapeutic relevance of the Nono–Kcnq2 axis. From a translational perspective, targeting Nono to restore its activity or developing pharmacological Nono agonists may represent innovative strategies for alleviating neuropathic pain. Future studies will focus on identifying small molecules or biologics that can selectively enhance Nono function, offering a promising direction for precision medicine interventions in chronic pain disorders.

Recently, scRNA-seq has revolutionized mechanistic dissection of neuropathic pain by decoding cellular and molecular heterogeneity within injured sciatic nerves and dorsal root ganglia (DRG), thereby elucidating the core pathophysiological mechanisms underlying the role of peripheral nervous system in pain progression. Specifically, peripheral nerve injury induces dynamic transcriptomic remodeling in defined DRG neuronal subpopulations, including ATF3/galanin-related injury-induced clusters and Fgf3^+^ neurons, and triggers persistent expansion of platelet-derived growth factor receptor beta (Pdgfrb)-positive fibroblasts and mural cells with sustained pronociceptive mediator secretion, collectively evidencing neuronal–non-neuronal coregulation of chronic pathogenesis [12,61,62]. By contrast, studies at the spinal cord level remain limited, and those available predominantly indicate that impaired oligodendrocyte differentiation undermines remyelination and perpetuates persistent pain [15]. To further investigate the role of Nono in pain, we employed scRNA-seq to characterize the cell types expressing Nono and to elucidate the temporal dynamics of spinal cord neuronal features associated with neuropathic pain. Through scRNA-seq, we identified multiple neuronal subtypes—Nrxn3^+^, Prph^+^, Plch2^+^, Lmo3^+^, Reln^+^, and others—each displaying distinct expression profiles at various stages following CCI injury. Nrxn3^+^, Plch2^+^, and Prph^+^ neurons were up-regulated to varying degrees on day 7 post-CCI, with Nrxn3^+^ and Plch2^+^ returning to baseline by day 21, while Prph remained persistently elevated. Furthermore, we detected a prominent co-enrichment of Nono and Kcnq2 in Nrxn3^+^ and Prph^+^ neurons. Subsequently, we employed spatial transcriptomics to identify and map the spatial localization of Nrxn3^+^ and Prph^+^ neurons, with Nrxn3^+^ neurons predominantly located in the spinal cord dorsal horn and Prph^+^ neurons in the anterior horn. Additional multicolor immunofluorescence staining showed increased Nono and Kcnq2 immunoreactivity in both RBFOX3^+^/Nrxn3^+^ and RBFOX3^+^/Prph^+^ neurons after Ad-Nono-OE treatment. Because viral reporter/tag-based tracing was not performed, these findings should be interpreted as supportive colocalization evidence rather than definitive proof of cell-subtype-specific viral transduction. Nrxn3, a member of the Neurexin family, functions as an adhesion molecule located at the presynaptic terminal, where it interacts with postsynaptic partners to establish and maintain synaptic connections between neurons [63]. At excitatory presynaptic terminals, Nrxn3 binds to postsynaptic ligands, such as leucine-rich repeat transmembrane neuronal proteins (LRRTMs) and glutamate Delta-1 receptor (GluD1), to stabilize the localization and clustering of AMPA receptors and ensure efficient glutamate transmission and proper synaptic function [64–67]. Prph is an intermediate filament protein predominantly found in neurons of the peripheral nervous system and certain central nervous system regions projecting to peripheral structures [68,69]. Prph expression is typically elevated following nerve injury or neurodegenerative diseases, reflecting neuronal stress responses and the activation of axonal repair processes, which contribute to nerve fiber stabilization and promote neuronal regeneration [70–72]. However, nerve regeneration led to the formation of abnormal regenerating nerves or neuromas, which amplify nociceptor hyperexcitability and drive the persistence of pain by altering the neuroimmune environment and engaging sympathetic nerve pathways [73–75].

Several limitations of this study should be noted. First, only male rats were included in the present study. Although this design helped reduce biological variability for mechanistic analyses, it limits the generalizability of our findings across sexes. Given that sex is an important biological variable in pain research, whether the Nono–Kcnq2 axis contributes similarly to neuropathic pain in female animals remains to be determined in future studies. Second, our study establishes the Nono–Kcnq2 axis as an important modulator of spinal neuronal excitability and neuropathic pain, but the upstream mechanisms leading to impaired Nono function after nerve injury remain unclear. VRK2 was identified through predictive analysis as a possible upstream regulator; however, it was not experimentally validated in the current study and should therefore be interpreted cautiously. Future studies should determine whether VRK2 directly regulates Nono phosphorylation and whether manipulation of VRK2 affects Kcnq2 expression, neuronal excitability, and pain-related behaviors. Third, although we have demonstrated Nono’s role in pain-related behaviors, neuronal excitability, and the phosphorylation of key postsynaptic receptor proteins, the specific phosphorylation sites on Nono that modulate these functions have not been identified and require further systematic investigation. Future studies using targeted mutagenesis, neuron-selective manipulation, and synaptic functional assays will be needed to define how site-specific phosphorylation events regulate synapse-related protein activity in neuropathic pain. Finally, although the Nono–Kcnq2 axis may have potential translational relevance, this work remains primarily mechanistic and preclinical. Further studies are required to determine whether this pathway can be therapeutically targeted in neuropathic pain.

In summary, we integrated proteomics, phosphoproteomics, and catTFRE analysis to demonstrate that CCI leads to reduced Nono activity, resulting in decreased Kcnq2 expression and increased neuronal excitability. Furthermore, scRNA-seq and spatial transcriptomics revealed that Nono and Kcnq2 colocalize in Nrxn3^+^ neurons in the dorsal horn and Prph^+^ neurons in the spinal cord ventral horn. These findings support a model in which CCI suppresses Nono expression or activity in distinct neuronal populations, impairing Kcnq2-mediated potassium signaling, thereby contributing to heightened neuronal excitability and neuropathic pain. Together, our results uncover a previously unrecognized Nono–Kcnq2 molecular axis critical for nociceptive regulation. Targeting this axis—either by pharmacological activation of Kcnq2 channels or genetic enhancement of Nono function—may represent a precision medicine strategy for neuropathic pain.

## Materials and Methods

### Animal and experimental design

Sprague–Dawley rats, male, aged 7 weeks and weighing between 150 and 170 g, were acquired from Henan Skobes Biotechnology Co., Ltd. (Certificate NO. SCXK[Yu]2020-0005, Anyang, China). Animals were kept in a specific pathogen-free animal laboratory, with controlled conditions. Each experimental group was assigned animals at random. The Guizhou University of Traditional Chinese Medicine Experimental Animal Ethics Committee approved all animal procedures (Approval No. 20240826003).

In Experiment 1, 53 rats were randomly assigned to the sham group (*n* = 23) or CCI group (*n* = 30). On day 7 after surgery, 15 rats from each group were randomly selected for multi-omics analyses and electrophysiological assessment. The remaining 15 rats in the CCI group and 8 rats in the sham group were maintained until day 21. At this time point, the remaining 15 CCI rats were further subjected to the multi-omics analyses, whereas the remaining 8 sham rats served only as behavioral controls. In Experiment 2, 32 rats were randomly assigned to the sham, CCI, CCI+Ad-NC, or CCI+Ad-Nono OE group (*n* = 8 per group) to assess the effects of Nono overexpression on pain hypersensitivity, Kcnq2 expression, and the electrophysiological properties of spinal neurons. In Experiment 3, 30 rats were randomly assigned to the sham, CCI+Ad-NC+Vehicle (Veh) group, CCI+Ad-NC+XE991 group, CCI+Ad-Nono-OE+Veh group, and CCI+Ad-Nono-OE+ XE991 group (*n* = 6 per group) to determine whether pharmacological inhibition of Kcnq2 reverses the analgesic effect induced by Nono overexpression.

### CCI model

The preparation method of the CCI model was referred to the previous literature [76]. Rats were deeply anesthetized with intraperitoneal anesthetization of pentobarbitone sodium and positioned prone. After incising the skin and dissecting the muscle, the left sciatic nerve was exposed and moderately ligated 4 times with 4-0 silk at 1-mm intervals. Sham rats were identically exposed except for ligation. Following surgery, animals were monitored until recovery from anesthesia and were checked daily for general condition and wound status during the postoperative period.

### Construction of Nono overexpression adenovirus and intrathecal injection

The rat Nono gene was cloned into the GV314 vector. Cloning was performed using the BamHI and AgeI restriction sites. The recombinant plasmid was used for adenoviral packaging, and the virus was purified and titrated by plaque formation assay, yielding a final titer of 1.5 × 10^10^ plaque-forming units (PFU)/ml. On postoperative day 1 after CCI surgery, rats received intrathecal adenoviral administration via implanted intrathecal catheters. The total viral dose was fixed at 1 × 10^9^ PFU per rat in both groups. Because of differences in viral titers, different stock volumes were required to achieve the same viral dose. Specifically, the Ad-NC virus stock had a titer of 1.0 × 10^11^ PFU/ml, requiring a total of 10 μl virus solution per rat, whereas the Ad-Nono-OE virus stock had a titer of 1.5 × 10^10^ PFU/ml, requiring a total of 66.7 μl virus solution per rat. To avoid excessive single-injection volume, the total injection volume was divided into 6 equal intrathecal injections administered over 3 consecutive days (2 injections per day), with each injection volume fixed at 11 μl. For the Ad-NC group, phosphate-buffered saline (PBS) was added to the virus solution to match the injection volume used in the Ad-Nono-OE group. Thus, both groups received identical total injection volumes and identical viral doses. MWT and TWL were measured to assess nociceptive sensitivity at baseline and on postoperative days 0, 3, 7, 14, and 21.

### XE991 administration

XE991 (HY-108577A, MedChemExpress, Monmouth Junction, USA), a selective Kcnq2/3-selective blocker, was intraperitoneally administered at 3 mg/kg before behavioral tests in the CCI+Ad-NC+XE991 and CCI+Ad-Nono-OE+XE991 groups, as previously described. The remaining groups received an equal volume of normal saline.

### Behavioral tests

MWT and TWL were evaluated to assess mechanical and thermal hyperalgesia using von Frey filaments and a plantar heat stimulator, respectively, at baseline and 3, 7, 11, 14, and 21 days following CCI surgery. MWT was assessed using calibrated von Frey filaments (0.008 to 300 g; Aesthesio, DanMic Global, San Jose, CA, USA) applied to the mid-plantar surface of the ipsilateral hind paw. Each filament was applied perpendicularly until slight bending was observed and held for 3 to 5 s. A brisk paw withdrawal, flinching, or licking was considered a positive response. MWT was defined as the minimum force that elicited withdrawal responses in at least 50% of repeated applications [77,78]. Individual MWT values (g) were ln-transformed before statistical analysis [79]. TWL was measured using a plantar heat stimulator (37570-001, Ugo Basile, Gemonio, Italy). A focused infrared heat source was applied to the mid-plantar surface to deliver a radiant thermal stimulus, and paw withdrawal latency was recorded upon withdrawal responses, including licking, lifting, or rapid withdrawal. Each rat was tested 5 times at 5- to 10-min intervals, and the mean value was used for statistical analysis.

### Sample collection and preparation

For the multi-omics analysis, randomly selected rats from the sham and CCI groups were deeply anesthetized at 7 and 21 days after CCI surgery. A laminectomy was then performed to expose the spinal cord, and ipsilateral L4 to L6 spinal cord tissues were rapidly dissected for subsequent analyses. In a separate set of experiments, spinal cord tissues for the Nono overexpression study were collected exclusively at 21 days after surgery using the same procedure.

### Proteomics analysis

Tissue samples were thoroughly washed with ice-cold PBS to remove residual blood and contaminants, followed by mechanical homogenization and lysis in a urea-based denaturing buffer containing protease and phosphatase inhibitors. Brief sonication was applied to facilitate cell disruption, and insoluble material was removed by high-speed centrifugation at 12,000 rpm for 10 min to obtain clarified protein extracts. Protein concentrations were measured using a Bradford assay. Protein integrity and sample loading consistency were evaluated by sodium dodecyl sulfate–polyacrylamide gel electrophoresis (SDS-PAGE; MA0461, Dalian Meilun Biotechnology Co., Ltd., Dalian, China) with Coomassie Brilliant Blue staining. For each sample, 200 μg of protein was reduced with 10 mM dithiothreitol (D9163-5G, Sigma-Aldrich, Burlington, USA) at 56 °C for 30 min and alkylated with 10 mM iodoacetamide (V900335-25g, Sigma-Aldrich) at room temperature in the dark for 30 min. The samples were combined with 200 μl of 8 M urea bicarbonate in a filter unit, vortexed, and centrifuged at 12,000 rpm for 20 min. The filters were washed with 200 μl of urea buffer and 200 μl of 50 mM NH_4_HCO_3_ buffer. The proteins were digested overnight with 4 μg of trypsin in 100 μl of 50 mM NH_4_HCO_3_ buffer at 37 °C. The resulting peptides were collected as a filtrate and vacuum-dried.

Proteome profiling was performed using a hybrid quadrupole-Orbitrap mass spectrometer (Orbitrap Exploris 480, Thermo Fisher Scientific) coupled to a nanoflow high-performance liquid chromatography system (EASY-nLC 1200, Thermo Fisher Scientific). Peptides were reconstituted in solvent A, loaded onto a trapping column, and separated on a 15-cm reversed-phase analytical column using a 120-min linear gradient of 4% to 100% solvent B at a flow rate of 600 nl/min. Solvent A consisted of 0.1% formic acid (A117-50, Thermo Fisher Scientific) in water, and solvent B consisted of 0.1% formic acid in 80% acetonitrile (A955-4-CASE, Thermo Fisher Scientific). Data were acquired in data-dependent acquisition mode. Full MS scans were collected in the Orbitrap at a resolution of 120,000 with an AGC target of3 × 10^6^ . MS/MS spectra were generated by higher-energy collisional dissociation with a normalized collision energy of 30% and acquired at a resolution of 7,500 with an AGC target of5 × 10^4^. Dynamic exclusion was enabled with an exclusion duration of 40 s. FAIMS Pro was applied during acquisition using compensation voltages of −45 and −65 V.

Proteomic data were processed using the iProteome analytical platform. Unsupervised hierarchical clustering was performed and visualized in R using the Pheatmap package. Functional enrichment analyses, including Gene Ontology and KEGG pathway annotations, were conducted with the DAVID database. Protein–protein interaction networks were constructed based on STRING database resources.

### Phosphoproteomics analysis

Protein extraction, SDS-PAGE, filter-aided sample preparation, and MS analysis were performed as previously detailed in the “Proteomics analysis” section. Tryptic peptides were enriched for phosphopeptides using the High-Select Fe-NTA kit (A32992, Thermo Fisher Scientific) according to the manufacturer’s instructions. Peptides were suspended in the kit’s binding/wash buffer provided by the kit and combined with the equilibrated resins. The peptide–resin mixture was left to incubate at room temperature for 30 min. The resins were then rinsed 3 times with binding/wash buffer and twice with water. The enriched peptides were eluted using the kit’s elution buffer and promptly dried with a SpeedVac at 30 °C for mass spectrometry analysis. The iProteome platform was utilized for data analysis. Differentially phosphorylated sites were identified using the criteria of fold change ≥ 2 and *P* < 0.05. Bioinformatic analyses were performed, including clustering of phosphorylated peptides, GO annotation, KEGG annotation, and protein–protein interaction (PPI) analysis.

### catTFRE analysis

catTFRE deploys biotinylated tandem-TF-consensus DNA probes to enrich endogenous TF complexes through sequence-specific chromatin affinity pulldown, circumventing antibody-dependent limitations of conventional methods [80]. Tissues were washed with ice-cold PBS and homogenized in Cytoplasmic Extraction Reagent I (NE-PER kit, 78835, Thermo Fisher Scientific) to obtain nuclear protein fractions. Nuclear extracts were quantified using the Bradford assay, and protein integrity was assessed by SDS-PAGE followed by Coomassie Blue staining.

Biotinylated catTFRE DNA probes (3 pmol) were immobilized on streptavidin-coated magnetic beads (Dynabeads M-280, 11206D, Thermo Fisher Scientific) and incubated with nuclear extracts under high-salt conditions (final NaCl concentration 200 to 250 mM) in the presence of ethylenediaminetetraacetic acid (EDTA, 10009617, Sinopharm Chemical Reagent Co., Ltd.) at 4 °C for 2 h. After incubation, beads were extensively washed with NETN buffer (100 mM NaCl, 20 mM Tris-Cl, 0.5 mM EDTA, and 0.5% Nonidet P-40, N8030, Solarbio Science and Technology Co., Ltd., Beijing, China) and PBS to remove nonspecifically bound proteins. Bound protein complexes were subjected to on-bead tryptic digestion in 50 mM ammonium bicarbonate at 37 °C overnight. Peptides were subsequently eluted using 50% acetonitrile containing 0.1% formic acid and vacuum-dried (Concentrator plus, Eppendorf, Hamburg, Germany).

Liquid chromatography–tandem mass spectrometry (LC–MS/MS) analysis was performed on a Q Exactive HF-X hybrid quadrupole–Orbitrap mass spectrometer coupled to an EASY-nLC 1200 system. Peptides were separated on a reversed-phase analytical column using a 150-min linear gradient of acetonitrile under nano-flow conditions and analyzed in data-dependent acquisition mode. Full MS scans were acquired in the Orbitrap at high resolution over an *m*/*z* range of 300 to 1,400, followed by higher-energy collisional dissociation of the most abundant precursor ions. Dynamic exclusion was applied to minimize repeated sequencing of identical ions.

Raw mass spectrometry data were processed using the iProteome cloud platform for qualitative and quantitative analysis. Hierarchical clustering was performed in R using the Pheatmap package.

Differentially expressed genes were identified using the criteria of fold change ≥ 1.2 and *P* < 0.05. Functional annotation and pathway enrichment analyses were conducted based on Gene Ontology and KEGG databases, and protein–protein interaction networks were constructed to characterize catTFRE-enriched TF–associated protein complexes.

### ScRNA-seq analysis

Spinal cord samples were minced and incubated in 1 ml of NST lysis buffer (0.1% Nonidet P-40, 10 mM Tris-HCl, 146 mM NaCl, 1 mM CaCl_2_, 21 mM MgCl_2_, and 40 U/ml RNase inhibitor, EO0382, Thermo Fisher Scientific) for 7 min to permeabilize cellular membranes and release nuclei. Subsequently, 1 ml of ST Wash buffer (10 mM Tris-HCl, 146 mM NaCl, 1 mM CaCl_2_, 21 mM MgCl_2_, 0.01% bovine serum albumin [BSA] [1000076, Miltenyi Biotec, Bergisch Gladbach, Germany], and 40 U/ml RNase inhibitor) was added, and the suspension was passed through a 40-μm cell strainer (352340, Corning Incorporated, Corning, USA). The cell sieve was rinsed with ST Wash buffer, and the filtrate was centrifuged for 5 min (500 *g*, 4 °C). Nuclei were initially resuspended in 5 ml of PBS with 1% BSA, centrifuged, and then resuspended in 100 μl of the same solution. The concentration of single-nucleus suspension was diluted to a concentration of 700 to 1,200 cells/μl using PBS with 1% BSA. The library was prepared following the manufacturer’s instructions using the Chromium Next GEM Single Cell 3′ Reagent Kits v3.1 (PN-1000268, 10× Genomics, Pleasanton, CA, USA) and sequenced on an Illumina sequencing platform (NovaSeq X Plus, Illumina Inc., San Diego, USA).

FASTQ files were processed using Cell Ranger (v7.0.1) and aligned to the mRatBN7.2 reference genome. The resulting UMI count matrix was analyzed using Seurat (v4) [81]. Low-quality nuclei were excluded using the following thresholds: <200 detected genes, <1,000 UMIs, log10GenesPerUMI <0.7, mitochondrial RNA UMIs >10%, or hemoglobin RNA UMIs >5%. Doublets were subsequently removed using the DoubletFinder package [82]. Data normalization was performed using NormalizeData, and highly variable genes were identified with FindVariableFeatures. Dimensionality reduction was conducted by principal component analysis (PCA), and 2-dimensional visualization was generated using UMAP. Cluster-enriched marker genes were identified with FindAllMarkers, whereas differentially expressed genes were determined using FindMarkers.

### Spatial transcriptomics analysis

Spinal cord samples were transversely sectioned, embedded in optimal cutting temperature (OCT) compound, and stored at −80 °C. Frozen tissue blocks were cut into 10-μm cryosections, mounted onto ST array, dehydrated with isopropanol for 1 min, and subsequently stained with H&E. Brightfield images were captured using a scanner (Pannoramic MIDI, 3D HISTECH Ltd., Budapest, Hungary) at 40× resolution. Following the manufacturer’s instructions for the Visium Spatial Gene Expression Slide & Reagents Kit (PN-1000184, 10× Genomics), the tissue sections underwent permeabilization, reverse transcription, cDNA amplification, and DNA library construction. Initially, the stained sections were permeabilized with permeabilization enzyme and added with Master Mix containing RT reagents to generate cDNAs tagged with spatial barcodes. Subsequently, Second Strand Master Mix was applied to synthesize the second-strand cDNAs on the slide. The spatial barcodes were encoded, and cDNAs were amplified using polymerase chain reaction to generate enough mass for library construction. Sequencing of the qualified DNA libraries was performed on the NovaSeq X Plus platform.

The FASTQ files and histology images were processed by SpaceRanger software (version 1.2.0), which employs STAR for genome alignment against mRatBN7.2 reference genome. The UMI count matrix was processed with the Seurat R package and normalized via SCTransform. Highly variable genes (HVGs) across single cells were identified using the method described in the literature [83]. PCA was then performed on the log-transformed gene-barcode matrices of the HVGs to reduce dimensionality. Cell clustering was conducted using graph-based clustering methods and visualized in a 2-dimensional space with UMAP. SPOTlight utilizes non-negative matrix factorization to integrate marker-guided scRNA-seq with spatial transcriptomics, enabling the deconvolution of cellular composition in Visium spots [84].

### Whole-cell patch-clamp recordings in SDH slices

Rats were anesthetized deeply and perfused transcardially with ice-cold, oxygenated cutting solution. The spinal column was quickly dissected and placed in an ice-cold, oxygenated cutting solution composed of 93 mM *N*-methyl-d-glucamine, 2.5 mM KCl, 1.2 mM NaH_2_PO_4_, 20 mM HEPES, 25 mM d-glucose, 30 mM NaHCO_3_, 10 mM MgSO_4_, 0.5 mM CaCl_2_, 5 mM sodium ascorbate, 3 mM sodium pyruvate, and 2 mM thiourea, with a pH of 7.3 to 7.4 and an osmolarity of 300 mOsm. Transverse spinal cord slices (300 μm) from the ipsilateral L4 to L6 segments were prepared using a vibratome (VT1200S, Leica Biosystems, Nussloch, Germany), incubated at 33 °C for 15 min in a cutting solution, and then transferred to a recovery solution with 93 mM NaCl, 2.5 mM KCl, 1.2 mM NaH_2_PO_4_, 20 mM HEPES, 25 mM d-glucose, 30 mM NaHCO_3_, 2 mM MgSO_4_, 2 mM CaCl_2_, 5 mM sodium ascorbate, 3 mM sodium pyruvate, and 2 mM thiourea (pH 7.3 to 7.4, 300 mOsm). The slices were kept at room temperature for 1 h before being transferred to an upright fluorescence microscope (BX51WI, Olympus Corporation, Tokyo, Japan) for recordings. All recordings were performed in oxygenated artificial cerebrospinal fluid (ACSF) under continuous perfusion at 33 °C using an in-line temperature controller. To maintain tissue viability, solutions were continuously aerated with a 95% O_2_ and 5% CO_2_ mixture. After initially localizing the SDH at 10× magnification, the objective was adjusted to 40× magnification to perform whole-cell patch-clamp recordings from SDH neurons. Patch pipettes were fabricated from borosilicate glass capillaries (BF150-86-10, Sutter Instrument, Novato, USA) using a micropipette puller (P-97, Sutter Instrument) to obtain a resistance of 3 to 5 MΩ. The pipette solution consisted of 135 mM K-gluconate, 10 mM NaCl, 10 mM HEPES, 0.5 mM EGTA, 10 mM Na_2_-phosphocreatine, 4 mM Mg-ATP, and 0.4 mM Na_2_-GTP, with the pH adjusted to 7.3 using KOH and an osmolarity of 290 to 300 mOsm. Recordings utilized a patch-clamp amplifier (Multiclamp 700B, Molecular Devices, Sunnyvale, USA) and a data acquisition system (DigiData 1440A, Molecular Devices), with signals low-pass filtered and digitized at 10 kHz. Clampex and Clampfit software (pCLAMP 10.6, Molecular Devices) were utilized for data acquisition and analysis. Only cells with acceptable recording quality (series resistance < 25 MΩ, input resistance > 300 MΩ, and leak current < 50 pA) were included in the analysis. Neurons were randomly selected for recording to avoid sampling bias.

### Immunofluorescence staining

Tissue blocks underwent dehydration using a graded ethanol series and followed by clearing in xylene. They were then infiltrated with paraffin wax at 60 °C in 3 steps. Sections were cut, mounted on slides, and baked at 60 °C for 3 h. Deparaffinization involved incubation in xylene and ethanol, followed by washing with distilled water. Antigen retrieval was conducted in 0.01 M EDTA buffer (pH 9.0) at 95 °C for 15 min. Endogenous peroxidase activity was inhibited using 3% hydrogen peroxide for 15 min. Sections were blocked with normal goat serum, followed by overnight incubation with primary antibodies at 4 °C, and a 50-min incubation with horseradish peroxidase (HRP)-conjugated secondary antibodies. The tyramide signal amplification fluorescent dye (RC0086Plus-45RM, Recordbio Biological Technology, Shanghai, China) was used for 5 min, followed by 25 min antibody elution at 95 °C. The labeling process was repeated for 4 rounds. Nuclei were stained with DAPI (4′,6-diamidino-2-phenylindole), and sections were mounted using antifade medium prior to fluorescence microscopy imaging. Primary antibodies were as follows: RBFOX3 (ab177487, Abcam, Cambridge, UK, 1:1,500), Nono (ab70335, Abcam, 1:2,000), Kcnq2 (ab22897, Abcam, 1:1,000), Prph (7399-1-AP, Proteintech, Chicago, USA, 1:2,000), and Nrxn3 (NBP1-88424, Novus Biologicals, Centennial, USA, 1:1,000).

### Western blot

Protein extraction from spinal cord tissues was performed using RIPA lysis buffer (MA0151, Meilun Biotechnology Co., Ltd.) with added protease and phosphatase inhibitors (MB12707, Meilun Biotechnology Co., Ltd.). Tissue samples were homogenized with a bead mill homogenizer (TissueLyser II, QIAGEN, Hilden, Germany), lysed on ice for 30 min, and centrifuged at 12,000 rpm for 5 min at 4 °C. Protein concentrations in the collected supernatant were quantified using a BCA protein assay kit (G3422, GBCBIO, Guangzhou, China). Protein samples (40 μg) were combined with 5× loading buffer, denatured by boiling for 10 min, and separated using SDS-PAGE in a vertical electrophoresis system (DYCZ-24DN, Beijing Liuyi Biotechnology Co., Ltd., Beijing, China). After electrophoresis, proteins were transferred to polyvinylidene difluoride (PVDF) membranes using a semi-dry transfer system (eBlot L1, Nanjing GenScript Biotech Co., Ltd., Nanjing, China). Membranes were blocked using 5% nonfat milk in TBST for 2 h at room temperature, followed by an overnight incubation at 4 °C with primary antibodies: anti-Nono (Ab70335, Abcam, 1:5,000), anti-Kcnq2 (Ab22897, Abcam, 1:1,000), and anti-GAPDH (60004-1-Ig, Proteintech, 1:10,000), all diluted in the blocking buffer. Following 5 TBST washes, membranes were incubated with HRP-conjugated secondary antibodies at room temperature for 2 h. Protein bands were then visualized using an enhanced chemiluminescence (ECL) detection system, captured with a chemiluminescence imaging system (SH-523, Shenhua Science Technology Co., Ltd., Huangzhou, China), and quantified via densitometry with Image-Pro Plus software. GAPDH served as the internal loading control.

### ChIP assay

ChIP assays were performed using spinal cord tissues collected from sham and CCI rats at 21 days post-surgery with the EpiQuik ChIP Kit (Epigentek, Farmingdale, NY, USA), according to the manufacturer’s instructions with minor optimization for spinal cord tissue. Briefly, spinal cord tissues were finely minced and homogenized in PBS to generate a tissue suspension. The suspension was centrifuged at 1,000 rpm for 5 min, washed with PBS to remove residual debris, and then cross-linked with 1% formaldehyde in PBS at room temperature for 10 min with gentle shaking. Cross-linking was quenched by adding 1.25 M glycine, followed by centrifugation and washing with ice-cold PBS. To reduce interference from spinal cord tissue components, including myelin-rich debris, the cell pellet was first resuspended in CP3A buffer and incubated on ice for 10 min. After brief vortexing, the suspension was centrifuged at 5,000 rpm for 5 min, and the supernatant was carefully discarded. The remaining nuclear pellet was then resuspended in CP3B buffer containing protease inhibitor cocktail and incubated on ice for 10 min with intermittent mixing. Chromatin was sheared by sonication on ice, and the lysate was centrifuged at 14,000 rpm for 10 min to remove insoluble debris. For immunoprecipitation, the chromatin-containing supernatant was diluted with CP4 buffer at a 1:1 ratio, and an aliquot was reserved as input DNA. Antibody-coated strip wells were prepared by incubating CP2-pretreated wells with either 4 μg of anti-Nono antibody (11058-1-AP, Proteintech) or normal mouse IgG as a negative control at room temperature for 90 min. Diluted chromatin samples were then added to the antibody-coated wells and incubated at room temperature for 90 min with gentle shaking. The wells were washed 6 times with CP1 buffer and once with 1× TE buffer. For reversal of cross-linking and DNA purification, proteinase K-containing CP5 buffer was added to both immunoprecipitated samples and input samples, followed by incubation at 65 °C for 15 min. CP6 buffer was then added, and samples were further incubated at 65 °C for 90 min to complete reversal of cross-linking. DNA was purified using the spin columns supplied with the kit and eluted in CP8 buffer. DNA enrichment at the Kcnq2 promoter was quantified by qPCR using AceQ qPCR SYBR Green Master Mix (Q111-02, Vazyme Biotech Co., Ltd., Nanjing, China). The primers were custom-designed to amplify the Kcnq2 promoter region containing the predicted Nono-binding site 2, which includes the AGTGAG motif corresponding to the reverse-complement CTCACT sequence identified in the motif analysis. The primer sequences were as follows: forward, 5′-AGGTAGGTGATGGTAAA-3′; reverse, 5′-CTGGGTAAGGGAGGAGG-3′. Input, IgG, and anti-Nono immunoprecipitated samples were analyzed in parallel. The qPCR amplification program was as follows: initial denaturation at 95 °C for 10 min, followed by 40 cycles of denaturation at 95 °C for 15 s and annealing/extension at 60 °C for 60 s. Melting curve analysis was subsequently performed at 95 °C for 15 s, 60 °C for 60 s, and 95 °C for 15 s. All reactions were performed with 3 technical replicates.

### Dual-luciferase reporter assay

HEK-293T cells were maintained at 37 °C with 5% CO₂ in DMEM containing 10% FBS and 1% penicillin–streptomycin. Cells were seeded at a density of 1 × 10^5^ cells per well in 12-well plates and transfected the following day using Lipofectamine 2000 (Invitrogen, USA) as per the manufacturer’s instructions. To evaluate the transcriptional regulation of the Kcnq2 promoter by Nono, the wild-type Kcnq2 promoter sequence was cloned into the pGL3 reporter vector to generate the pGL3-Kcnq2 promoter construct. To assess the requirement of the predicted Nono-binding motif, the core AGTGAG motif within the Kcnq2 promoter, corresponding to the reverse-complement CTCACT motif identified in the motif analysis, was mutated to GACAGA to generate the pGL3-Kcnq2 promoter-Mut2 construct. In addition, Nono expression plasmids were constructed using pcDNA3.1, including wild-type Nono, a phospho-deficient mutant Nono-T433A/T455A (Nono-AA), and a phospho-mimetic mutant Nono-T433D/T455D (Nono-DD). In the Nono-AA construct, threonine residues at positions 433 and 455 were substituted with alanine to prevent phosphorylation, whereas in the Nono-DD construct, these residues were substituted with aspartic acid to mimic constitutive phosphorylation. For reporter assays, cells were cotransfected with the indicated pcDNA3.1-based expression plasmids, pGL3-based reporter plasmids, and pRL-TK as an internal control. The main plasmid combinations included pcDNA3.1 with pGL3-Basic, pcDNA3.1-Nono with pGL3-Basic, pcDNA3.1 with pGL3-Kcnq2 promoter, pcDNA3.1-Nono with pGL3-Kcnq2 promoter, pcDNA3.1-Nono-AA or pcDNA3.1-Nono-DD with pGL3-Kcnq2 promoter, and corresponding combinations with pGL3-Kcnq2 promoter-Mut2. Cells were lysed 48 h after transfection, and luciferase activity was measured using the Dual Luciferase Reporter Gene Assay Kit (RG027, Beyotime, Shanghai, China). Luminescence signals were detected using a multifunctional microplate reader (SuPerMax 3100, Shanghai Flash Spectrum Biological Technology Co., Ltd., Shanghai, China). Firefly luciferase activity was normalized to Renilla luciferase activity, and the relative luciferase activity was used to assess Kcnq2 promoter activation.

### Statistical analysis

Data were expressed as mean ± standard deviation (SD). Normality was assessed using the Shapiro–Wilk test, and homogeneity of variance was verified through Levene’s test. For comparisons involving more than 2 groups, data meeting the assumptions of normality and homogeneity of variance were analyzed by analysis of variance followed by Tukey’s multiple comparisons test for pairwise comparisons. This approach was applied to experiments involving multiple groups, including behavioral assays, Western blot, ChIP, and dual-luciferase reporter assays.

For some behavioral data that markedly deviated from normality, the Kruskal–Wallis test was used. For 2-group comparisons, such as electrophysiological data, an unpaired Student’s *t* test was used when data satisfied parametric assumptions. Statistical significance was defined as *P* < 0.05.

## Data Availability

The scRNA-seq and spatial transcriptomic datasets generated in this study have been deposited in the Gene Expression Omnibus (GEO) database under accession numbers GSE304729 and GSE304826, respectively. The proteomics, phosphoproteomics, and catTFRE datasets have been deposited in the iProX database under accession number IPX0013325000.

## References

[1] Cao B, Xu Q, Shi Y, Zhao R, Li H, Zheng J, Liu F, Wan Y, Wei B. Pathology of pain and its implications for therapeutic interventions. Signal Transduct Target Ther. 2024;9(1):155. 38851750 10.1038/s41392-024-01845-w PMC11162504

[2] Burke D, Fullen BM, Stokes D, Lennon O. Neuropathic pain prevalence following spinal cord injury: A systematic review and meta-analysis. Eur J Pain. 2017;21(1):29–44. 27341614 10.1002/ejp.905

[3] Song Z, Sun Y, Liu P, Ruan H, He Y, Yin J, Xiao C, Ma J, Yu Y, Wang S, et al. Terahertz wave alleviates comorbidity anxiety in pain by reducing the binding capacity of nanostructured glutamate molecules to GluA2. Research. 2024;7:0535. 39664293 10.34133/research.0535 PMC11633831

[4] Finnerup NB, Kuner R, Jensen TS. Neuropathic pain: From mechanisms to treatment. Physiol Rev. 2021;101(1):259–301. 32584191 10.1152/physrev.00045.2019

[5] Bannister K, Sachau J, Baron R, Dickenson AH. Neuropathic pain: Mechanism-based therapeutics. Annu Rev Pharmacol Toxicol. 2020;60(1):257–274. 31914896 10.1146/annurev-pharmtox-010818-021524

[6] Inoue K, Tsuda M. Microglia in neuropathic pain: Cellular and molecular mechanisms and therapeutic potential. Nat Rev Neurosci. 2018;19(3):138–152. 29416128 10.1038/nrn.2018.2

[7] Marty-Lombardi S, Lu S, Ambroziak W, Schrenk-Siemens K, Wang J, DePaoli-Roach AA, Hagenston AM, Wende H, Tappe-Theodor A, Simonetti M, et al. Neuron-astrocyte metabolic coupling facilitates spinal plasticity and maintenance of inflammatory pain. Nat Metab. 2024;6(3):494–513. 38443593 10.1038/s42255-024-01001-2 PMC10963271

[8] Liu X, Zhou L. Long-term potentiation at spinal C-fiber synapses: A target for pathological pain. Curr Pharm Des. 2015;21(7):895–905. 25345608 10.2174/1381612820666141027115949

[9] Rosner J, de Andrade DC, Davis KD, Gustin SM, Kramer JLK, Seal RP, Finnerup NB. Central neuropathic pain. Nat Rev Dis Primers. 2023;9(1):73. 38129427 10.1038/s41572-023-00484-9 PMC11329872

[10] Fiore NT, Debs SR, Hayes JP, Duffy SS, Moalem-Taylor G. Pain-resolving immune mechanisms in neuropathic pain. Nat Rev Neurol. 2023;19(4):199–220. 36859719 10.1038/s41582-023-00777-3

[11] Jiang B, Ding T, Guo C, Bai X, Cao D, Wu X, Sha W, Jiang M, Wu L, Gao Y. NFAT1 orchestrates spinal microglial transcription and promotes microglial proliferation via c-MYC contributing to nerve injury-induced neuropathic pain. Adv Sci. 2022;9(27): Article e2201300. 10.1002/advs.202201300 PMC9507349 35892263

[12] Wang K, Wang S, Chen Y, Wu D, Hu X, Lu Y, Wang L, Bao L, Li C, Zhang X. Single-cell transcriptomic analysis of somatosensory neurons uncovers temporal development of neuropathic pain. Cell Res. 2021;31(8):904–918. 33692491 10.1038/s41422-021-00479-9 PMC8324866

[13] Yang Y, Wen J, Zheng B, Wu S, Mao Q, Liang L, Li Z, Bachmann T, Bekker A, Tao Y. CREB participates in paclitaxel-induced neuropathic pain genesis through transcriptional activation of Dnmt3a in primary sensory neurons. Neurotherapeutics. 2021;18(1):586–600. 33051852 10.1007/s13311-020-00931-5 PMC8116406

[14] Tang F, Li J, Qi L, Liu D, Bo Y, Qin S, Miao Y, Yu K, Hou W, Li J, et al. A pan-cancer single-cell panorama of human natural killer cells. Cell. 2023;186(19):4235–4251. 37607536 10.1016/j.cell.2023.07.034

[15] Li D, Yang K, Li J, Xu X, Gong L, Yue S, Wei H, Yue Z, Wu Y, Yin S. Single-cell sequencing reveals glial cell involvement in development of neuropathic pain via myelin sheath lesion formation in the spinal cord. J Neuroinflammation. 2024;21(1):213. 39217340 10.1186/s12974-024-03207-3 PMC11365210

[16] Shi H, He Y, Zhou Y, Huang J, Maher K, Wang B, Tang Z, Luo S, Tan P, Wu M, et al. Spatial atlas of the mouse central nervous system at molecular resolution. Nature. 2023;622(7983):552–561. 37758947 10.1038/s41586-023-06569-5 PMC10709140

[17] Rao A, Barkley D, Franca GS, Yanai I. Exploring tissue architecture using spatial transcriptomics. Nature. 2021;596(7871):211–220. 34381231 10.1038/s41586-021-03634-9 PMC8475179

[18] Zhang D, Chen Y, Wei Y, Chen H, Wu Y, Wu L, Li J, Ren Q, Miao C, Zhu T, et al. Spatial transcriptomics and single-nucleus RNA sequencing reveal a transcriptomic atlas of adult human spinal cord. Elife. 2024;12:RP92046. 38289829 10.7554/eLife.92046 PMC10945563

[19] Longo SK, Guo MG, Ji AL, Khavari PA. Integrating single-cell and spatial transcriptomics to elucidate intercellular tissue dynamics. Nat Rev Genet. 2021;22(10):627–644. 34145435 10.1038/s41576-021-00370-8 PMC9888017

[20] Yao Z, van Velthoven CTJ, Kunst M, Zhang M, McMillen D, Lee C, Jung W, Goldy J, Abdelhak A, Aitken M, et al. A high-resolution transcriptomic and spatial atlas of cell types in the whole mouse brain. Nature. 2023;624(7991):317–332. 38092916 10.1038/s41586-023-06812-z PMC10719114

[21] Cable DM, Murray E, Zou LS, Goeva A, Macosko EZ, Chen F, Irizarry RA. Robust decomposition of cell type mixtures in spatial transcriptomics. Nat Biotechnol. 2022;40(4):517–526. 33603203 10.1038/s41587-021-00830-w PMC8606190

[22] Moncada R, Barkley D, Wagner F, Chiodin M, Devlin JC, Baron M, Hajdu CH, Simeone DM, Yanai I. Integrating microarray-based spatial transcriptomics and single-cell RNA-seq reveals tissue architecture in pancreatic ductal adenocarcinomas. Nat Biotechnol. 2020;38(3):333–342. 31932730 10.1038/s41587-019-0392-8

[23] Li S, Damonte VM, Chen C, Wang GX, Kebschull JM, Yamaguchi H, Bian W, Purmann C, Pattni R, Urban AE, et al. Hyperexcitable arousal circuits drive sleep instability during aging. Science. 2022;375(6583):eabh3021. 35201886 10.1126/science.abh3021 PMC9107327

[24] Alles S, Smith PA. Etiology and pharmacology of neuropathic pain. Pharmacol Rev. 2018;70(2):315–347. 29500312 10.1124/pr.117.014399

[25] Latremoliere A, Woolf CJ. Central sensitization: A generator of pain hypersensitivity by central neural plasticity. J Pain. 2009;10(9):895–926. 19712899 10.1016/j.jpain.2009.06.012 PMC2750819

[26] Ji RR, Nackley A, Huh Y, Terrando N, Maixner W. Neuroinflammation and central sensitization in chronic and widespread pain. Anesthesiology. 2018;129(2):343–366. 29462012 10.1097/ALN.0000000000002130 PMC6051899

[27] Jiang L, Liu N, Zhao F, Huang B, Kang D, Zhan P, Liu X. Discovery of GluN2A subtype-selective N-methyl-d-aspartate (NMDA) receptor ligands. Acta Pharm Sin B. 2024;14(5):1987–2005. 38799621 10.1016/j.apsb.2024.01.004 PMC11119548

[28] Chou TH, Epstein M, Fritzemeier RG, Akins NS, Paladugu S, Ullman EZ, Liotta DC, Traynelis SF, Furukawa H. Molecular mechanism of ligand gating and opening of NMDA receptor. Nature. 2024;632(8023):209–217. 39085540 10.1038/s41586-024-07742-0 PMC11376105

[29] Dupuis JP, Nicole O, Groc L. NMDA receptor functions in health and disease: Old actor, new dimensions. Neuron. 2023;111(15):2312–2328. 37236178 10.1016/j.neuron.2023.05.002

[30] Zhang YY, Liu F, Fang ZH, Li YL, Liao HL, Song QX, Zhou C, Shen JF. Differential roles of NMDAR subunits 2A and 2B in mediating peripheral and central sensitization contributing to orofacial neuropathic pain. Brain Behav Immun. 2022;106:129–146. 36038077 10.1016/j.bbi.2022.08.010

[31] Foster KA, McLaughlin N, Edbauer D, Phillips M, Bolton A, Constantine-Paton M, Sheng M. Distinct roles of NR2A and NR2B cytoplasmic tails in long-term potentiation. J Neurosci. 2010;30(7):2676–2685. 20164351 10.1523/JNEUROSCI.4022-09.2010 PMC2840640

[32] Hildebrand ME, Xu J, Dedek A, Li Y, Sengar AS, Beggs S, Lombroso PJ, Salter MW. Potentiation of synaptic GluN2B NMDAR currents by Fyn kinase is gated through BDNF-mediated disinhibition in spinal pain processing. Cell Rep. 2016;17(10):2753–2765. 27926876 10.1016/j.celrep.2016.11.024

[33] Kim Y, Cho H, Ahn YJ, Kim J, Yoon YW. Effect of NMDA NR2B antagonist on neuropathic pain in two spinal cord injury models. Pain. 2012;153(5):1022–1029. 22424878 10.1016/j.pain.2012.02.003

[34] Buonarati OR, Hammes EA, Watson JF, Greger IH, Hell JW. Mechanisms of postsynaptic localization of AMPA-type glutamate receptors and their regulation during long-term potentiation. Sci Signal. 2019;12(562):eaar6889. 30600260 10.1126/scisignal.aar6889 PMC7175813

[35] Liang Z, Li L, Bai L, Gao Y, Qiao Y, Wang X, Yv L, Xu J. Spinal nerve transection-induced upregulation of SAP97 via promoting membrane trafficking of GluA1-containing AMPA receptors in the dorsal horn contributes to the pathogenesis of neuropathic pain. Neurobiol Dis. 2024;194: Article 106471. 38461868 10.1016/j.nbd.2024.106471

[36] Taylor BK, Sinha GP, Donahue RR, Grachen CM, Moron JA, Doolen S. Opioid receptors inhibit the spinal AMPA receptor ca (2+) permeability that mediates latent pain sensitization. Exp Neurol. 2019;314:58–66. 30660616 10.1016/j.expneurol.2019.01.003 PMC6559354

[37] Zhang D, Hua Z, Li Z. The role of glutamate and glutamine metabolism and related transporters in nerve cells. CNS Neurosci Ther. 2024;30(2): Article e14617. 38358002 10.1111/cns.14617 PMC10867874

[38] Goh GY, Huang H, Ullman J, Borre L, Hnasko TS, Trussell LO, Edwards RH. Presynaptic regulation of quantal size: K+/H+ exchange stimulates vesicular glutamate transport. Nat Neurosci. 2011;14(10):1285–1292. 21874016 10.1038/nn.2898 PMC3183113

[39] Pietrancosta N, Djibo M, Daumas S, El Mestikawy S, Erickson JD. Molecular, structural, functional, and pharmacological sites for vesicular glutamate transporter regulation. Mol Neurobiol. 2020;57(7):3118–3142. 32474835 10.1007/s12035-020-01912-7 PMC7261050

[40] Waxman SG, Zamponi GW. Regulating excitability of peripheral afferents: Emerging ion channel targets. Nat Neurosci. 2014;17(2):153–163. 24473263 10.1038/nn.3602

[41] Rose K, Ooi L, Dalle C, Robertson B, Wood IC, Gamper N. Transcriptional repression of the M channel subunit Kv7.2 in chronic nerve injury. Pain. 2011;152(4):742–754. 21345591 10.1016/j.pain.2010.12.028 PMC3071978

[42] Huang Z, Lujan R, Kadurin I, Uebele VN, Renger JJ, Dolphin AC, Shah MM. Presynaptic HCN1 channels regulate Cav3.2 activity and neurotransmission at select cortical synapses. Nat Neurosci. 2011;14(4):478–486. 21358644 10.1038/nn.2757 PMC3068302

[43] Noh W, Pak S, Choi G, Yang S, Yang S. Transient potassium channels: Therapeutic targets for brain disorders. Front Cell Neurosci. 2019;13:265. 31263403 10.3389/fncel.2019.00265 PMC6585177

[44] Zhang F, Gadotti VM, Souza IA, Chen L, Zamponi GW. BK potassium channels suppress Cavalpha2delta subunit function to reduce inflammatory and neuropathic pain. Cell Rep. 2018;22(8):1956–1964. 29466724 10.1016/j.celrep.2018.01.073

[45] Brown DA, Passmore GM. Neural KCNQ (Kv7) channels. Br J Pharmacol. 2009;156(8):1185–1195. 19298256 10.1111/j.1476-5381.2009.00111.x PMC2697739

[46] Wang H, Hu S, Zhang S, Song Y, Wang X, Wang L, Li Y, Yu Y, Liu H, Liu D, et al. KCNQ channels in the mesolimbic reward circuit regulate nociception in chronic pain in mice. Neurosci Bull. 2021;37(5):597–610. 33900570 10.1007/s12264-021-00668-x PMC8099961

[47] Wu Z, Toro G, Xu G, Dang D, Prater C, Yang Q. Paclitaxel inhibits KCNQ channels in primary sensory neurons to initiate the development of painful peripheral neuropathy. Cells. 2022;11(24):4067. 36552832 10.3390/cells11244067 PMC9776748

[48] Oyama M, Watanabe S, Iwai T, Tanabe M. Selective inhibition of A-fiber-mediated excitatory transmission underlies the analgesic effects of KCNQ channel opening in the spinal dorsal horn. Neuropharmacology. 2024;254: Article 109994. 38750803 10.1016/j.neuropharm.2024.109994

[49] Cheng HM, Penninger JM. Transcriptional mechanisms underlying neuropathic pain: DREAM, transcription factors and future pain management? Expert Rev Neurother. 2002;2(5):677–689. 19810984 10.1586/14737175.2.5.677

[50] Edelmayer RM, Brederson J, Jarvis MF, Bitner RS. Biochemical and pharmacological assessment of MAP-kinase signaling along pain pathways in experimental rodent models: A potential tool for the discovery of novel antinociceptive therapeutics. Biochem Pharmacol. 2014;87(3):390–398. 24300134 10.1016/j.bcp.2013.11.019

[51] Kong E, Li Y, Ma P, Zhang Y, Ding R, Hua T, Yang M, Yuan H. Lyn-mediated glycolysis enhancement of microglia contributes to neuropathic pain through facilitating IRF5 nuclear translocation in spinal dorsal horn. J Cell Mol Med. 2023;27(12):1664–1681. 37132040 10.1111/jcmm.17759 PMC10273059

[52] Tao Y, Wang Q, Li X, Liu Y, Sun R, Xu H, Zhang M, Li S, Yang L, Wang H, et al. Spinal-specific super enhancer in neuropathic pain. J Neurosci. 2023;43(49):8547–8561. 37802656 10.1523/JNEUROSCI.1006-23.2023 PMC10711714

[53] Wei Y, Luo H, Yee PP, Zhang L, Liu Z, Zheng H, Zhang L, Anderson B, Tang M, Huang S, et al. Paraspeckle protein NONO promotes TAZ phase separation in the nucleus to drive the oncogenic transcriptional program. Adv Sci. 2021;8(24): Article e2102653. 10.1002/advs.202102653 PMC8693076 34716691

[54] Knott GJ, Bond CS, Fox AH. The DBHS proteins SFPQ, NONO and PSPC1: A multipurpose molecular scaffold. Nucleic Acids Res. 2016;44(9):3989–4004. 27084935 10.1093/nar/gkw271 PMC4872119

[55] Zhang S, Cooper JA, Chong YS, Naveed A, Mayoh C, Jayatilleke N, Liu T, Amos S, Kobelke S, Marshall AC, et al. NONO enhances mRNA processing of super-enhancer-associated GATA2 and HAND2 genes in neuroblastoma. EMBO Rep. 2023;24(2): Article e54977. 36416237 10.15252/embr.202254977 PMC9900351

[56] Zhang Y, Cui D, Huang M, Zheng Y, Zheng B, Chen L, Chen Q. NONO regulates B-cell development and B-cell receptor signaling. FASEB J. 2023;37(4): Article e22862. 36906291 10.1096/fj.202201909RR

[57] Belur NR, Bustos BI, Lubbe SJ, Mazzulli JR. Nuclear aggregates of NONO/SFPQ and A-to-I-edited RNA in Parkinson’s disease and dementia with Lewy bodies. Neuron. 2024;112(15):2558–2580. 38761794 10.1016/j.neuron.2024.05.003 PMC11309915

[58] Li W, Karwacki-Neisius V, Ma C, Tan L, Shi Y, Wu F, Shi YG. Nono deficiency compromises TET1 chromatin association and impedes neuronal differentiation of mouse embryonic stem cells. Nucleic Acids Res. 2020;48(9):4827–4838. 32286661 10.1093/nar/gkaa213 PMC7229820

[59] Liu X, Zheng J, Qi S, Shen Q. NONO regulates cortical neuronal migration and postnatal neuronal maturation. Neurosci Bull. 2019;35(6):1097–1101. 31502212 10.1007/s12264-019-00428-y PMC6864022

[60] Mircsof D, Langouet M, Rio M, Moutton S, Siquier-Pernet K, Bole-Feysot C, Cagnard N, Nitschke P, Gaspar L, Znidaric M, et al. Mutations in NONO lead to syndromic intellectual disability and inhibitory synaptic defects. Nat Neurosci. 2015;18(12):1731–1736. 26571461 10.1038/nn.4169 PMC5392243

[61] Villa-Hernandez S, Walker JV, Hore Z, Fedele L, Zebochin I, Li Y, Davis H, Kanda T, Shimizu F, Taams LS, et al. A role for fibroblast and mural cell subsets in a nerve ligation model of neuropathic pain? Brain Behav Immun. 2025;129:15–29. 40381746 10.1016/j.bbi.2025.05.012 PMC7618384

[62] Zhang C, Hu M, Wang X, Cui X, Liu J, Huang Q, Cao X, Zhou F, Qian J, He S, et al. scRNA-sequencing reveals subtype-specific transcriptomic perturbations in DRG neurons of Pirt^EGFPf^ mice in neuropathic pain condition. Elife. 2022;11: Article e76063. 36264609 10.7554/eLife.76063 PMC9584610

[63] Oku S, Siddiqui TJ. A GPI-anchored Neurexin 3 proteoform mediates dendritic inhibition. Neuron. 2022;110(13):2041–2044. 35797957 10.1016/j.neuron.2022.06.005

[64] Gomez AM, Traunmuller L, Scheiffele P. Neurexins: Molecular codes for shaping neuronal synapses. Nat Rev Neurosci. 2021;22(3):137–151. 33420412 10.1038/s41583-020-00415-7 PMC7612283

[65] Lloyd BA, Han Y, Roth R, Zhang B, Aoto J. Neurexin-3 subsynaptic densities are spatially distinct from Neurexin-1 and essential for excitatory synapse nanoscale organization in the hippocampus. Nat Commun. 2023;14(1):4706. 37543682 10.1038/s41467-023-40419-2 PMC10404257

[66] Sudhof TC. Synaptic neurexin complexes: A molecular code for the logic of neural circuits. Cell. 2017;171(4):745–769. 29100073 10.1016/j.cell.2017.10.024 PMC5694349

[67] Zhang R, Jiang H, Liu Y, He G. Structure, function, and pathology of Neurexin-3. Genes Dis. 2023;10(5):1908–1919. 37492720 10.1016/j.gendis.2022.04.008 PMC10363586

[68] Alkaslasi MR, Piccus ZE, Hareendran S, Silberberg H, Chen L, Zhang Y, Petros TJ, Le Pichon CE. Single nucleus RNA-sequencing defines unexpected diversity of cholinergic neuron types in the adult mouse spinal cord. Nat Commun. 2021;12(1):2471. 33931636 10.1038/s41467-021-22691-2 PMC8087807

[69] Lin H, He Z, Ebert S, Schornig M, Santel M, Nikolova MT, Weigert A, Hevers W, Kasri NN, Taverna E, et al. NGN2 induces diverse neuron types from human pluripotency. Stem Cell Rep. 2021;16(9):2118–2127. 10.1016/j.stemcr.2021.07.006 PMC8452516 34358451

[70] Liang Y, Tong F, Zhang L, Zhu L, Li W, Huang W, Zhao S, He G, Zhou Y. iTRAQ-based proteomic analysis discovers potential biomarkers of diffuse axonal injury in rats. Brain Res Bull. 2019;153:289–304. 31539556 10.1016/j.brainresbull.2019.09.004

[71] Reid AJ, Welin D, Wiberg M, Terenghi G, Novikov LN. Peripherin and ATF3 genes are differentially regulated in regenerating and non-regenerating primary sensory neurons. Brain Res. 2010;1310:1–7. 19913522 10.1016/j.brainres.2009.11.011

[72] Sabbatini D, Raggi F, Ruggero S, Seguso M, Mandrioli J, Cagnin A, Briani C, Toffanin E, Gizzi M, Fortuna A, et al. Evaluation of peripherin in biofluids of patients with motor neuron diseases. Ann Clin Transl Neurol. 2021;8(8):1750–1754. 34264016 10.1002/acn3.51419 PMC8351396

[73] Davies AJ, Kim HW, Gonzalez-Cano R, Choi J, Back SK, Roh SE, Johnson E, Gabriac M, Kim M, Lee J, et al. Natural killer cells degenerate intact sensory afferents following nerve injury. Cell. 2019;176(4):716–728. 30712871 10.1016/j.cell.2018.12.022 PMC6418410

[74] Xie W, Strong JA, Zhang J. Active nerve regeneration with failed target reinnervation drives persistent neuropathic pain. ENeuro. 2017;4(1):ENEURO.0008-17.2017. 10.1523/ENEURO.0008-17.2017 PMC5290455 28197545

[75] Zhu A, Shen L, Xu L, Chen W, Huang Y. Wnt5a mediates chronic post-thoracotomy pain by regulating non-canonical pathways, nerve regeneration, and inflammation in rats. Cell Signal. 2018;44:51–61. 29339085 10.1016/j.cellsig.2018.01.017

[76] Chen P, Wang C, Gong Q, Chai Y, Chen Y, Song C, Wu Y, Wang L. Alterations of endogenous pain-modulatory system of the cerebral cortex in the neuropathic pain. IScience. 2023;26(5): Article 106668. 37168579 10.1016/j.isci.2023.106668 PMC10165265

[77] Yang F, Luo W, Sun W, Wang Y, Wang J, Yang F, Li C, Wei N, Wang X, Guan S, et al. SDF1-CXCR4 signaling maintains central post-stroke pain through mediation of glial-neuronal interactions. Front Mol Neurosci. 2017;10:226. 28785202 10.3389/fnmol.2017.00226 PMC5519565

[78] Deuis JR, Dvorakova LS, Vetter I. Methods used to evaluate pain behaviors in rodents. Front Mol Neurosci. 2017;10:284. 28932184 10.3389/fnmol.2017.00284 PMC5592204

[79] Choi GJ, Kang H, Lee OH, Ahn EJ, White FA, Cho YJ, Baek CW, Jung YH, Kwon JW. Effectiveness of maturity of *Rubus occidentalis* on hyperalgesia induced by acidic saline injection in rats. BMC Complement Med Ther. 2022;22(1):12. 35016667 10.1186/s12906-021-03491-z PMC8751266

[80] Ding C, Chan DW, Liu W, Liu M, Li D, Song L, Li C, Jin J, Malovannaya A, Jung SY, et al. Proteome-wide profiling of activated transcription factors with a concatenated tandem array of transcription factor response elements. Proc Natl Acad Sci USA. 2013;110(17):6771–6776. 23553833 10.1073/pnas.1217657110 PMC3637693

[81] Butler A, Hoffman P, Smibert P, Papalexi E, Satija R. Integrating single-cell transcriptomic data across different conditions, technologies, and species. Nat Biotechnol. 2018;36(5):411–420. 29608179 10.1038/nbt.4096 PMC6700744

[82] McGinnis CS, Murrow LM, Gartner ZJ. DoubletFinder: Doublet detection in single-cell RNA sequencing data using artificial nearest neighbors. Cell Syst. 2019;8(4):329–337. 30954475 10.1016/j.cels.2019.03.003 PMC6853612

[83] Macosko EZ, Basu A, Satija R, Nemesh J, Shekhar K, Goldman M, Tirosh I, Bialas AR, Kamitaki N, Martersteck EM, et al. Highly parallel genome-wide expression profiling of individual cells using nanoliter droplets. Cell. 2015;161(5):1202–1214. 26000488 10.1016/j.cell.2015.05.002 PMC4481139

[84] Elosua-Bayes M, Nieto P, Mereu E, Gut I, Heyn H. SPOTlight: Seeded NMF regression to deconvolute spatial transcriptomics spots with single-cell transcriptomes. Nucleic Acids Res. 2021;49(9): Article e50. 33544846 10.1093/nar/gkab043 PMC8136778

