# Supplementary material for: Multi-Omics Analysis Reveals Nono–Kcnq2 Regulation of Neuronal Excitability in Chronic Constriction Injury-Induced Neuropathic Pain
Source: Research (Wash D C). 2026 Jul 14;9:1366. doi: 10.34133/research.1366 (PMC13365582; doi:10.34133/research.1366)
Supplement: Supplementary 1 — Figs. S1 to S4 [file research.1366.f1.docx]

**Supplemental information**

**Multi-omics analysis reveals Nono-Kcnq2 regulation of neuronal excitability in** **neuropathic pain**

Peng Chen, Jing Wu, Shaoshuai Tang, Qian Gong, Chen Wang, Wenjing Wang, Yuanhua Wu, Ting Tang, Ruixi Luo, Zhibing Wu, Zhaoyu Qin, and Long Wang


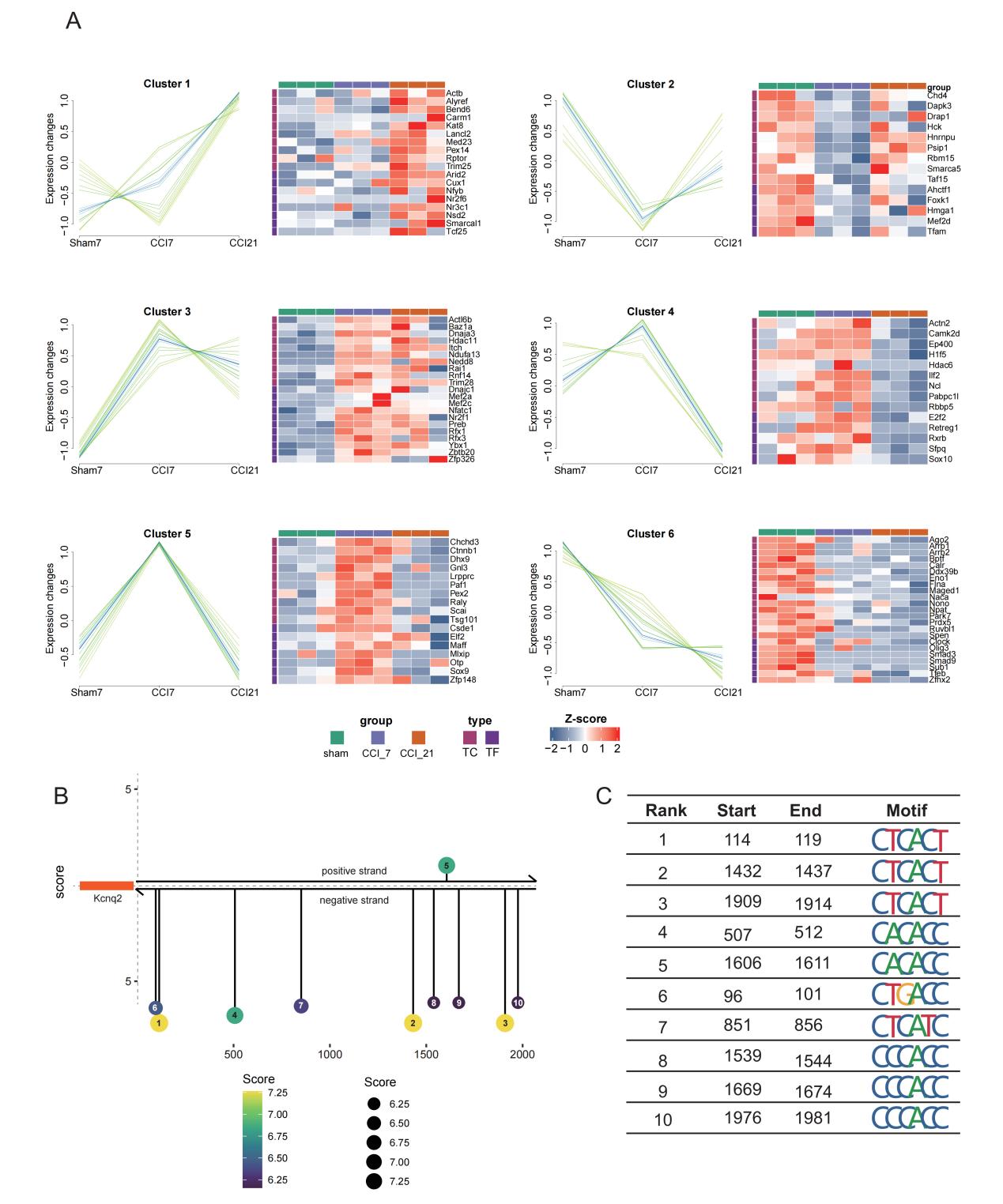


**Figure S1. Expression patterns of TFRE TFs and TCs across sham, CCI7 and CCI21**

Related to Figure 4.

1. Line charts and heat maps depicting the expression patterns of TFRE TFs and TCs across sham, CCI7 and CCI21. (B) Predicted Nono regulatory motifs in the 2kb upstream sequence of Kcnq2. (C) Potential Nono regulatory motif sites and patterns in the Kcnq2 promoter region.


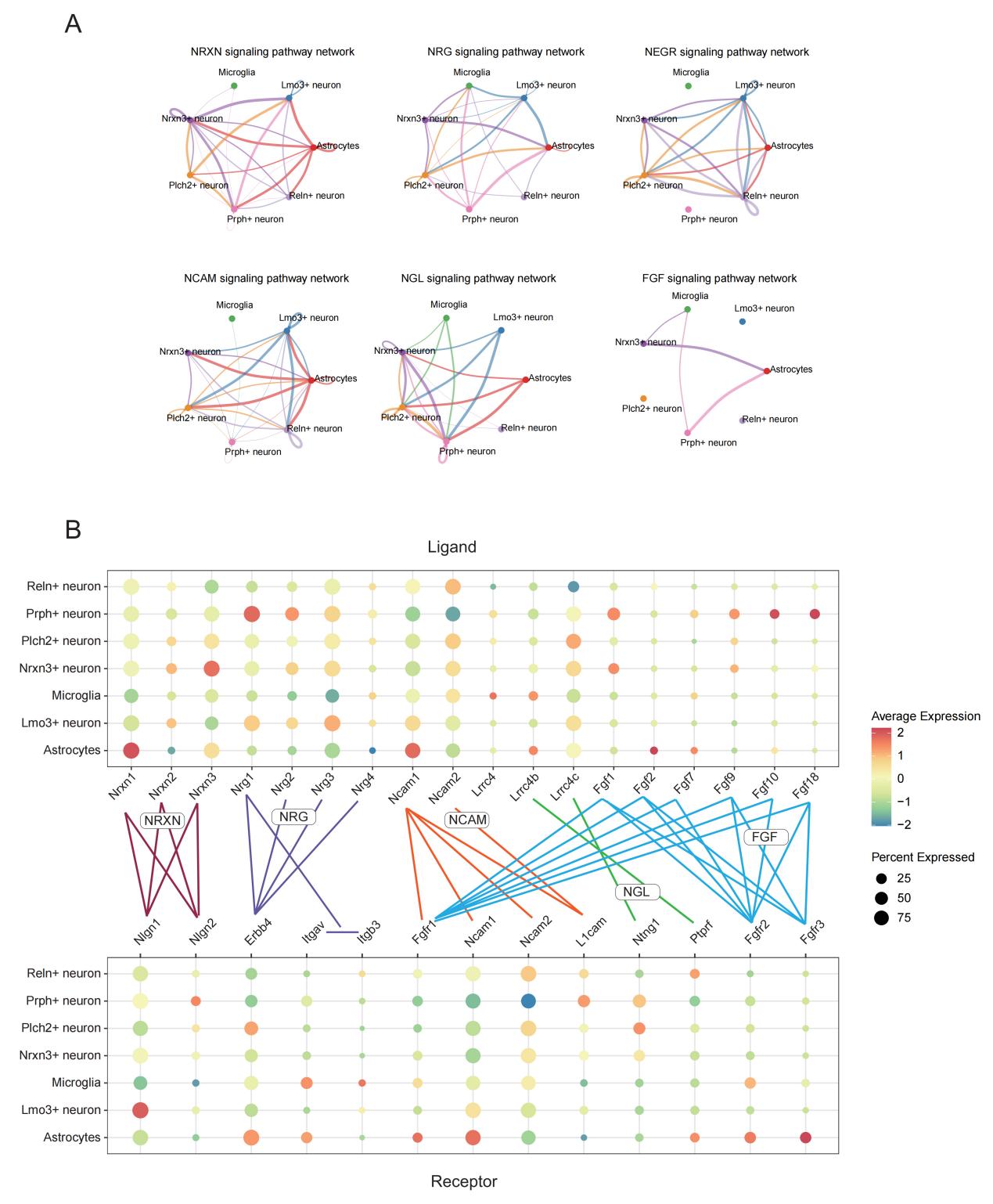


**Figure S**2. **CellChart analysis of intercellular communication among different neuronal subgroups, microglia, and astrocyte**

Related to Figure 6.

1. Network plots illustrating predicted signaling interactions across neuronal subtypes, microglia, and astrocyte. (B) Ligand-receptor expression heatmap and interaction details for selected signaling pathways.


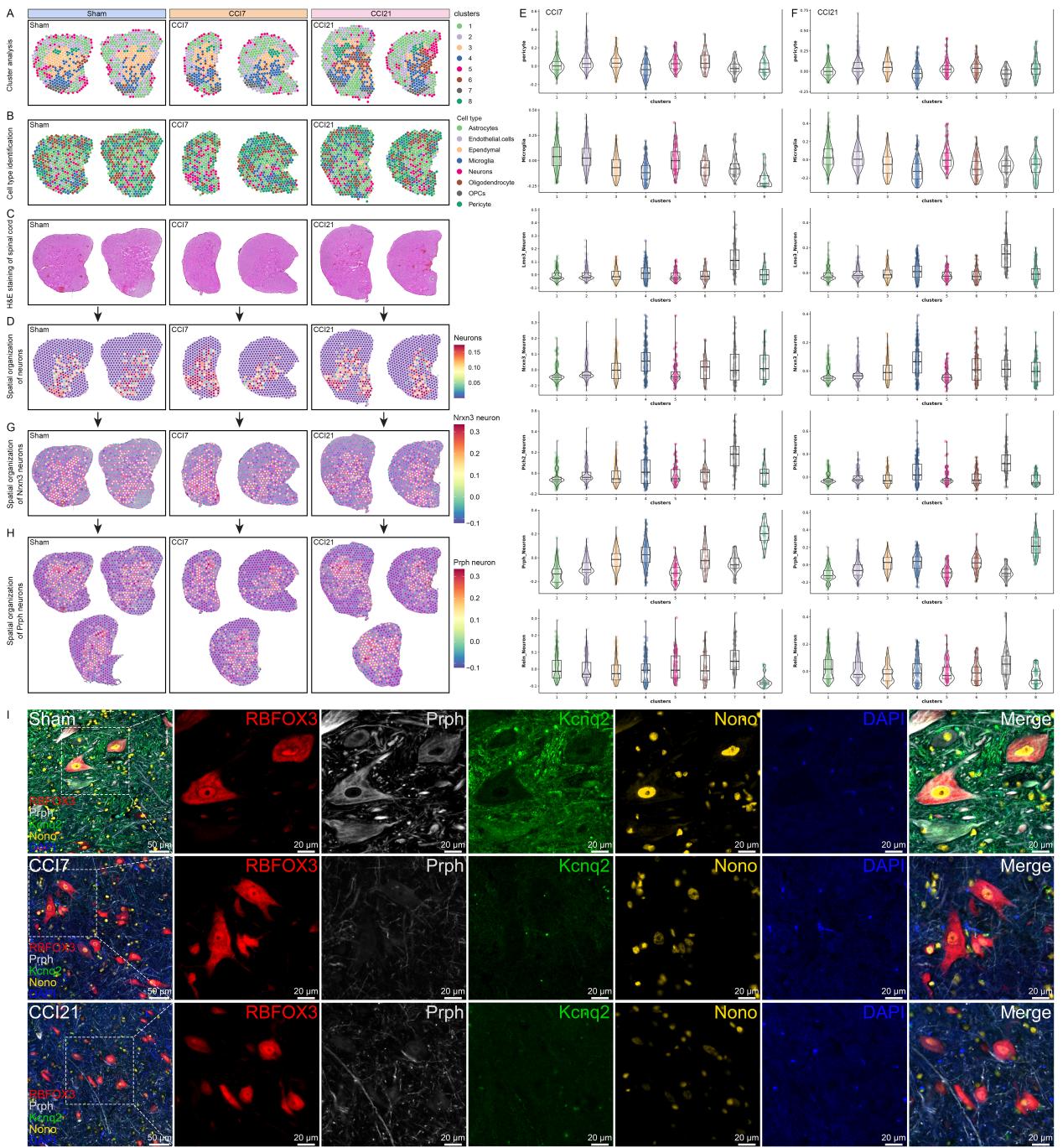


**Figure S3. Cellular composition and distribution of the spinal cord in neuropathic pain illustrated by spatial transcriptomics**

Related to Figure 7.

1. UMAP plot showing the distribution of clusters in spatial transcriptomics. (B) Spatial distribution of different cell types across the sham, CCI7 and CC21 group. (C) H&E staining of ipsilateral spinal cord tissues. (D) Spatial neuronal distribution across the sham, CCI7 and CC21 group. (E-F) Violin plot showing expression levels of different cell types across clusters. (G-H) Spatial distribution of Prph^+^ (G) and Nrxn3^+^ (H) neuron across the sham, CCI7 and CC21 group. (I) Immunofluorescence analysis confirming the co-localization of Nono and Kcnq2 in Prph^+^ neurons.


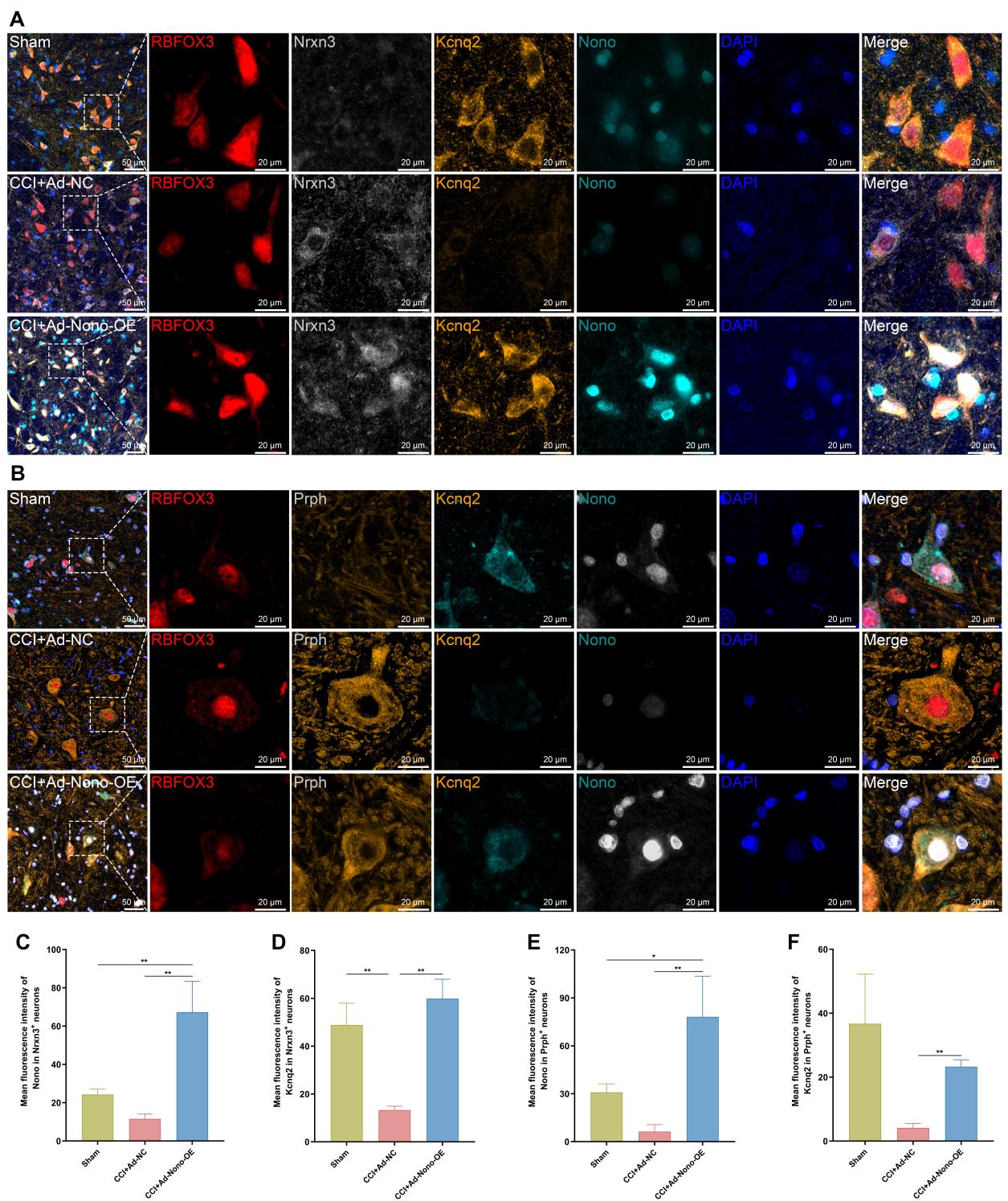


Fig. S4 Immunofluorescence analysis showing the co-localization of Nono and Kcnq2 in Nrxn3^+^ and Prph^+^ neurons across Sham, CCI+Ad-NC, and CCI+Ad-Nono-OE groups.

Related to Figure 8.

(A) The co-localization of Nono and Kcnq2 in Nrxn3^+^ neurons. (B) The co-localization of Nono and Kcnq2 in Prph^+^ neurons. (C-D) Quantitative analysis of Nono and Kcnq2 co-localization in Nrxn3⁺ and Prph⁺ neurons. **P* < 0.05, ***P* < 0.01 vs. the corresponding control group.
